# Body surface temperatures as biomarkers of physiological environmental adaptation in wild birds and mammals

**DOI:** 10.1111/brv.70085

**Published:** 2025-10-02

**Authors:** Paul Jerem, L. Michael Romero

**Affiliations:** ^1^ Groningen Institute for Evolutionary Life Sciences, University of Groningen 9700 CC Groningen The Netherlands; ^2^ Department of Biology Tufts University 200 College Ave, Robinson Hall Medford MA 02155 USA

**Keywords:** body temperature, surface temperature, physiology, thermoregulation, metabolism, stress, immune response, non‐invasive, infrared thermography, thermal imaging

## Abstract

The ability of individuals to cope with their environment, and therefore the likelihood that they survive and pass on their genes (i.e. fitness), is largely determined by physiological state. Tracking physiological state in wild animals, however, is challenging. Predominant techniques rely on capture and invasive procedures, restricting research to trappable species and individuals. Additionally, natural behaviours are interrupted, results may be affected by surgery or carrying apparatus, and welfare constraints restrict repeated sampling. Also, the leading non‐invasive alternative – faecal sampling – cannot detect rapid physiological changes. Thermal imaging offers an increasingly popular option for studying physiological state in homeothermic endotherms (birds and mammals). The method resolves many of the above concerns and can infer both fast and slow underlying physiological changes from body surface temperature dynamics. Nonetheless, the generalisability of results across settings and populations remains unclear because systematic synthesis is lacking. Correspondingly, important knowledge gaps may be currently overlooked for the same reason. To address these deficits, we performed a systematic review of research linking endotherm body surface temperatures and the four main physiological functions expected to influence surface temperatures – thermoregulation, metabolism, stress and immune responses. We combined outcomes into consensus profiles to ascertain whether responses are generalisable. We also evaluated article publication metrics, study subjects, and methods to characterise research trends and identify approaches most likely to drive progress. Consensus profiling suggested thermoregulatory, metabolic and acute stress (up to 3 min from stressor onset) body surface temperature responses are likely to be broadly generalisable. By contrast, body surface temperature dynamics during immune activation likely depend on discrete ranges of environmental conditions. However, the reviewed literature demonstrates that we still lack sufficient understanding of the mechanistic processes connecting body surface temperatures with underlying physiology. Therefore, further development of methods for inferring physiology from body surface temperatures in natural environments will require combinations of detailed laboratory validations and confirmatory field studies. Such research would also benefit from greater rigour than is evident in the currently available literature, in terms of routinely validating physiological challenges, avoiding use of stress‐inducing methods, analysing life‐history stage and sex differences, investigating effects of both challenge increase and decrease, and assessing responses across all possible thermoregulatory states. Assuming these knowledge gaps can be filled and technical challenges overcome, inferring physiology in the wild using thermal imaging will present a host of valuable eco‐evolutionary research opportunities surpassing those available with invasive or integrating techniques.

## I. INTRODUCTION

The ability of individuals to cope with their environment, and therefore the likelihood that they survive and pass on their genes, is largely determined by individual state. Physiological state is especially important in this respect, as physiological processes are dynamically adjusted to maximise fitness in response to both rhythmic (e.g. diurnal, seasonal; Kumar 2017) and unpredictable (e.g. food availability, predation risk; Romero & Wingfield, 2016) environmental variation. Consequently, understanding physiological state is key to uncovering why some individuals prosper while others perish, and so to revealing which populations might be at risk of extinction (Wingfield, 2008; Fefferman & Romero, 2013).

Nevertheless, investigating physiological state in wild animals is challenging, predominantly relying on techniques involving capture and handling and/or invasive procedures. For example, the recent *Philosophical Transactions of the Royal Society B* special issue *Measuring physiology in free living animals* was entirely devoted to applications of attached or implanted loggers (Hawkes, Fahlman & Sato, 2021). Also, blood sampling for doubly‐labelled water and plasma glucocorticoids remain ‘gold standards’ for assessing metabolic rate and physiological stress in natural environments (Romero & Wingfield, 2016; Speakman & Hambly, 2016). Similarly, many eco‐immunological assays typically require serological analysis (Downs & Stewart, 2014). Although such techniques are undoubtedly useful, and have yielded considerable insight into the ways wild animals use physiological processes to adapt to changing environments (Tomlinson *et al*., 2014; Garnier & Graham, 2014; Wilmers *et al*., 2015; Romero & Wingfield, 2016; Elliott, 2016; Ohmer *et al*., 2021), they have a number of critical shortcomings. As well as raising ethical concerns applicable to all invasive procedures used in animal research (Rollin, 2012), confining investigations to trappable species and individuals can limit generalisability and introduce bias (Garamszegi, Eens & Török, 2009; Stuber *et al*., 2013). Additionally, natural behaviours are interrupted, and subsequent physiology, behaviour and performance may all be altered as a result of capture and handling (van Oers & Carere, 2007; Lynn, Prince & Phillips, 2010), the burden of carrying apparatus (Casper, 2009; McMahon *et al*., 2011), or after‐effects of surgery (Hawkins, 2004). Welfare constraints on repeated invasive sampling also restrict efforts to track physiological state over time and establish response phenotypes, which are essential for linking physiological responses with fitness. Accordingly, the need for new methods that overcome these issues is clear. This demand has brought about the development of a range of non‐invasive techniques in recent years, principally targeting isotopes, metabolites, pathogens or genetic material in faeces or urine (Bourne *et al*., 2019; Palme, 2019; Schilling, Mazzamuto & Romeo, 2022). But, while sampling excreta is comparatively easy and may help avoid interfering with natural behaviours, it can only ever provide integrated measures over a time period defined by the subject's defecation/urination frequency (Goymann, 2005). Consequently, with minimum integration periods usually lasting at least a few hours (Palme *et al*., 2005), short‐term physiological changes are unlikely to be detected.

An alternative approach with the ability to detect both fast and slow physiological changes exploits associations between physiological processes and body temperature. Among endothermic homeotherms (species that generally maintain a stable body temperature, i.e. birds and mammals), body temperature dynamics have been linked with both short‐ and longer‐term physiological processes of particular relevance to fitness, including thermoregulation (Tan & Knight, 2018), metabolism (Refinetti, 2020), stress (Oka, 2018) and immune responses (Evans, Repasky & Fisher, 2015). Crucially, the advent of low‐cost, portable infrared thermal‐imaging cameras now permits non‐invasive, high‐frequency measurement of body surface temperatures from free‐living animals, using techniques similar to wildlife photography or filmmaking (Havens & Sharp, 2015). As a result, thermal imaging offers a potentially powerful new method for inferring endotherm physiological state in the wild, overcoming welfare and practical limitations of invasive methods, and offering sufficient temporal resolution to detect even sub‐second physiological fluctuations.

Given such promise, research output relating body surface temperatures to physiological state has grown in recent years across a range of disciplines including fundamental physiology, ecology, conservation, veterinary medicine, animal husbandry, psychology and welfare (McCafferty, 2013; Tattersall, 2016; Rekant *et al*., 2016; Travain & Valsecchi, 2021; Mota‐Rojas *et al*., 2022; Zheng *et al*., 2022). However, outcome consistency and generalisability (and therefore ease of application) across settings and populations remains unclear because systematic synthesis is lacking. Correspondingly, critical knowledge gaps may be currently overlooked for the same reason. To address these deficits as they pertain to investigating physiological state in wild birds and mammals, we performed a systematic review of research linking endotherm body surface temperatures and the four main physiological functions expected to influence surface temperatures: thermoregulation, metabolism, stress and immune responses. We combined outcomes into consensus profiles (*sensu* Dickens & Romero, 2013) to ascertain whether responses are generalisable. In addition, we evaluated article publication metrics, study subjects, and methods to characterise research trends and identify approaches most likely to drive progress.

## II. METHODS

### (1) Literature search

To examine current evidence linking endotherm body surface temperature to thermoregulation, metabolism, stress and immune responses, we created a database by systematically reviewing relevant literature. We used the *Web of Science* Core Collection to generate our list of candidate papers. Our search targeted all mammal and bird studies within the collection mentioning synonyms for body surface temperature and any of the four physiological functions listed above [search specification followed Grames *et al*. (2019); see online Supporting Information, Appendix S1, for details].

### (2) Study screening

Inclusion criteria were first applied to titles and abstracts. Full texts were then obtained for papers meeting our inclusion criteria, or where we lacked sufficient information to make a judgement. The inclusion criteria were then reapplied to the full texts to confirm eligibility using a PICO framework (Frampton, Livoreil & Petrokofsky, 2017), which focuses research questions by identifying target *Populations*, *Interventions*, *Comparisons* and *Outcomes*. Included articles were judged to report primary research addressing the question: *What is the relationship between body surface temperature and thermoregulatory, metabolic, stress or immune physiology?* Specific PICO components are shown in Table 1. Accordingly, eligible papers investigated non‐human endotherms; examined effects of thermoregulatory, metabolic, stress, or immune challenges; compared challenge with no‐challenge controls or different types or levels of challenge; and assessed surface temperature outcomes of challenge exposure. Preliminary results indicated our initial database was dominated by research using laboratory rodents (170 out of 346 papers categorised as potentially eligible). Given our interest is towards outcomes among wild animals occupying natural environments, we avoided heavy bias towards likely non‐representative species by excluding laboratory strain rat and mouse papers from our analyses. Similarly, inbred laboratory populations of zebra finch (*Taeniopygia guttata*) could also be considered non‐representative of their wild counterparts. However, as only one included paper used this species, this situation could not generate excessive bias, therefore this study was not excluded.

**Table 1:** Populations, Interventions, Comparisons and Outcomes (PICO) components used to define systematic review inclusion criteria.

Population	Non‐human endotherms (birds and mammals)	

Intervention	Challenge type	Definition

	Thermoregulation	Exposure to variation in environmental temperatures
	Metabolism	Exposure to situations associated with energetic consequences beyond routine exertion (e.g. moult, pregnancy, food restriction, or seasonal temperature change)
	Stress	Exposure to situations triggering sympathetic–adrenal–medullary or hypothalamic–pituitary–adrenal pathways (e.g. capture/restraint, social agonism)
	Immune	Exposure to pathogens, or stimulation of immune challenge *via* associated pyrogenic compounds (e.g. lipopolysacharrides, interleukins or prostaglandins)

Comparators	Absence, or differing types or levels of challenge	

Outcomes	Surface temperature effects of challenges	

### (3) Data extraction

We first separated included papers into functional groupings according to the challenge used (thermoregulation, metabolism, stress, immune response). Then, to quantify changes in the level of interest in this research over time, we calculated annual publication rates for each functional grouping. Next, to gain insight into the subjects (e.g. taxon, origin, life stage) and methods used (e.g. experimental or observational approach, acute or chronic exposure, surface type assessed, validations undertaken), we extracted the descriptive data listed in Table 2 from each included paper. Outcome‐related data were extracted differently depending on study type (Table 3). Where a paper fulfilled more than one level of a data class (e.g. it involved multiple species, populations, or stressors), or where analyses were separated (e.g. by environmental condition – below, within, above thermoneutral zone, or by pharmaceutical treatment dose) each category level was counted as an independent ‘study’ in our analyses. Therefore, sample sizes are dependent on the variable(s) analysed, and are in some cases larger than the number of included papers. For example, when analysing experimental exposure type (acute or chronic) in the thermoregulation group, one paper reported results of separate acute and chronic timescale tests. Consequently, for that analysis, the number of experimental papers (*N* = 42) was expanded by one (to give 43 studies), due to the splitting of the paper reporting acute and chronic tests into two studies (see Table 5A). For clarity, we use the term ‘paper’ to refer to whole publications, and ‘study’ where splitting occurred (i.e. when describing results) or is an implied possibility (i.e. in methodological descriptions, or during broad discussion). The effect of the splitting process is especially apparent in the outcomes analyses, where papers were split by study species, subject origin, subject life stage, study type, exposure type and target body surface region, as well as when analyses were separated as described above. The maximum and mean numbers of studies split from individual papers in the outcomes analyses were: Thermoregulation: maximum = 42, mean ± SD = 3.91 ± 5.57; Metabolism: maximum = 4, mean ± SD = 1.79 ± 1.12; Stress: maximum = 9, mean ± SD = 2.69 ± 2.10; Immune response: maximum = 5, mean ± SD 2.13 ± 1.12. It should be noted that domesticated animals of different strains/breeds used within the same paper were not counted as separate populations, as inter‐strain/breed differences are unlikely to be applicable in wild species. Also, where data appeared to be shared between papers (e.g. breeding‐success metrics for the same species from the same year at the same location), one paper was selected at random from the group, and the others were excluded.

**Table 2:** Descriptive data extracted from included studies.

Class	Attribute	Values
Subject	Taxonomic group	*Bird*, *Mammal*
	Origin	*Captive bred*, *Wild‐caught*, *Wild*
	Life stage	*Juvenile*, *Adult*
Methods	Study type	*Observational*, *Experimental*
	Exposure type	*Acute* (≤ 4 h), *Chronic* (≥ 4 h)
	Surface type	*Skin* (or scales), *Eye*, *Hair*, *Feather*, *Horn*, *Casque*, *Bill*, *Claw*, *Mixed*
	Insulation	*Insulated*, *Uninsulated*
	Sex differences analysed	*Yes*, *No*
	Physiological validation of challenge attempted	*Yes*, *No*
	Potential for methods used to induce confounding thermal physiological stress response (assessed only for baseline measurements in stress studies)	*High* (subject exposed to handling, restraint or (<1 m) experimenter approach during temperature measurement), *Low* (subject not exposed to handling, restraint or (<1 m) experimenter approach during temperature measurement), *Unclear* (methods description lacks sufficient detail to judge above experiences)
	Relationship between environmental temperatures and species thermoneutral zone (where thermoneutral zone data were available)	*Below*, *Within*, *Above*
	Measurement device used to record body surface temperature	*Digital thermometer*, *iButton*, *Thermocouple*, *Thermistor*, *Thermistor telemetry*, *Temperature logger*, *Infrared thermometer*, *Thermal imaging*

**Table 3:** Outcome‐related data extracted from included studies.

Study type	Outcome	Value(s)
Observational	Significant relationship between surface temperature and challenge	*Yes*, *No*
Experimental between‐group comparison	Significant surface temperature difference between challenge and control experimental groups	*Yes*, *No*
Experimental within‐individual comparison	(*a*) Immediate (within 1 min) significant surface temperature response to exposure onset relative to baseline (studies with measurement frequency ≤1 min only)	*Increase*, *Decrease*, *None*
	(*b*) Significant difference in surface temperature from baseline 3 min after exposure onset (linear interpolation used to provide estimates where necessary and possible, minimum measurement frequency = 5 min)	*No difference*, *Below*, *Above*
	(*c*) Significant difference in surface temperature from baseline 15 min after exposure onset (linear interpolation used to provide estimates where necessary and possible, minimum measurement frequency = 5 min)	*No difference*, *Below*, *Above*
	(*d*) Significant difference in surface temperature from baseline more than 15 min after exposure onset (if response direction changed over time, only direction of largest difference from baseline was recorded)	*No difference*, *Below*, *Above*
	(*e*) Response end time (body surface temperatures returned to baseline values for >2 time series measurements), where experiment end time is >15 min	*Hours after challenge onset*
	(*f*) Final post‐challenge surface temperature measurement timing if no response end time was observed, where experiment end time is >15 min	*Hours after challenge onset*
All study types	Direction of the predominant relationship between body surface temperature and comparator across all data presented (where relationships were variable over the course of an experiment, proportion of conditions under which the predominant relationship held was also noted as a number with the range 0–1).	*Positive*, *Negative*, *No relationship*

### (4) Consensus profiles

We assessed whether body surface temperature responses are generalisable using consensus profiling – a method of synthesising outcome direction (e.g. increase, decrease or no relationship) across studies, taking into account study quality (Dickens & Romero, 2013). To obtain consensus profiles of body surface temperature responses for the various outcome measures described above, we first ranked included studies according to the appropriateness of their study design in generating data relevant to outcomes in wild animals. Ranking followed Dickens & Romero (2013) and was based on the attribute scoring scheme presented in Table 4.

**Table 4:** Scoring scheme used to rank studies according to the appropriateness of their design in generating data relevant to outcomes in wild animals. Statistical power was determined by assigning study sample sizes (per treatment group) into quartiles based on a histogram of all included studies within a functional grouping (i.e. thermoregulation, metabolism, stress or immune response).

Attribute	Score (attribute specific)			
	1	2	3	4
Type of study	Observational	Experimental	–	–
Study population	Captive	Wild‐caught	Wild	–
Treatment comparator (thermoregulation and metabolism analyses)	Artificial	Real/simulation	–	–
Treatment comparator (stress and immune response analyses)	Pharmacological	Artificial	Real/simulation	–
Statistical power	*N* in 1st quartile	*N* in 2nd quartile	*N* in 3rd quartile	*N* in 4th quartile

For each study, attribute scores were then summed (resulting in a score ranging from 5 to 14), and study rank within the category group (thermoregulation, metabolism, stress, immune response) determined according to its score. To calculate consensus body surface temperature profiles for each category outcome, we multiplied each response outcome (arbitrarily assigned as decrease/below = 1, no relationship or difference = 2, increase/above = 3; chosen to avoid complications of multiplying and dividing by 0) by the proportion of conditions in which the relationship held (to penalise for inconsistent outcomes; see Table 3) and then multiplied by study rank (to weight outcomes according to study design). Lastly, we divided summed weighted response outcomes by the sum of all study ranks to provide a single final consensus profile value, and generated 95% confidence intervals from the mean response. Consensus profiles were only calculated when relevant data were reported by ≥5 studies.

### (5) Data visualisation

All data visualisation was performed using R. v4.4.0 (R Core Team, 2024) and *ggplot2* v3.5.1 (Wickham, 2016).

## III. RESULTS AND DISCUSSION

### (1) Systematic review

Our search identified 9625 unique articles, of which 141 papers met the inclusion criteria (Fig. S1, Database S1). Two full‐text publications were removed from the included group because they shared data with other included papers, leaving 139 included papers. From these 139 papers, 68 were included in the thermoregulation analyses, 14 in the metabolism analyses, 35 in the stress analyses and 24 in the immune response analyses (two papers were included in both thermoregulation and metabolism categories).

### (2) Publication rates

**Fig. 1:**
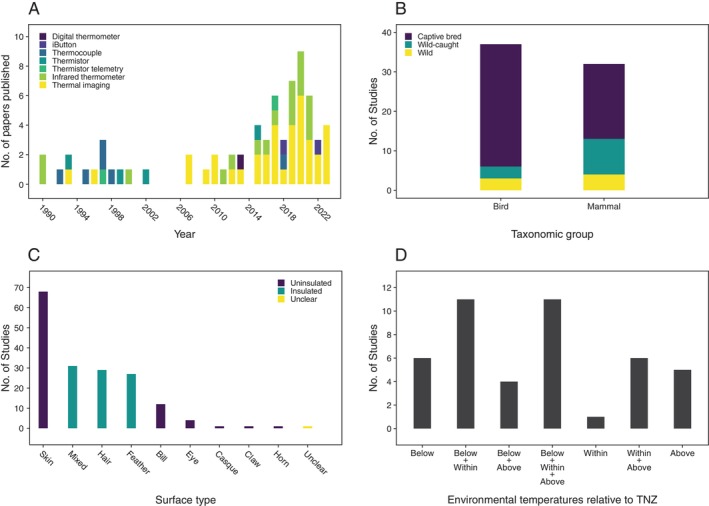
Numbers of included papers/studies (see Section II.3) investigating relationships between body surface temperatures and thermoregulation by (A) body surface temperature measurement method, grouped by year of publication, (B) subject population type, grouped by taxon, (C) insulation level of surface from which temperatures were measured, grouped by surface type and (D) relationship between environmental temperatures experienced during data collection and subject thermoneutral zone (TNZ) for species where such data were available. Thermoneutral zone categories separated by plus signs indicate mixed study conditions (e.g. Below+Above means conditions were both below and above the thermoneutral zone).

**Fig. 2:**
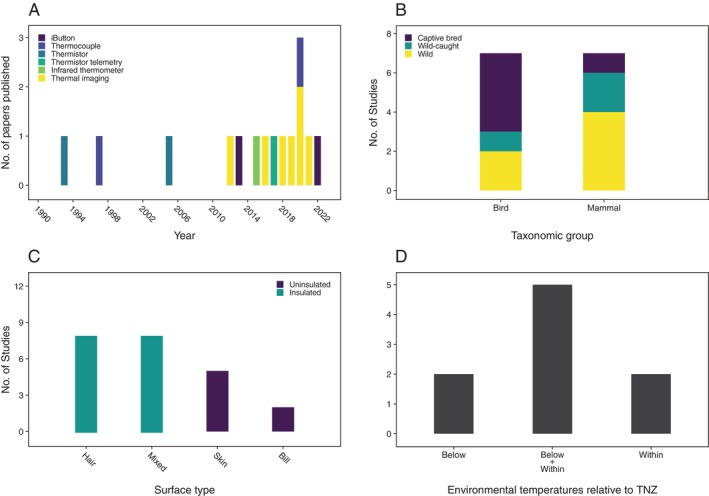
Numbers of included papers/studies (see Section II.3) investigating relationships between body surface temperatures and metabolism by (A) body surface temperature measurement method, grouped by year of publication, (B) subject population type, grouped by taxon, (C) insulation level of surface from which temperatures were measured, grouped by surface type and (D) relationship between environmental temperatures experienced during data collection and subject thermoneutral zone (TNZ) for species where such data were available Thermoneutral zone categories separated by plus signs indicate mixed study conditions (e.g. Below+Within means conditions were both below and within the thermoneutral zone).

**Fig. 3:**
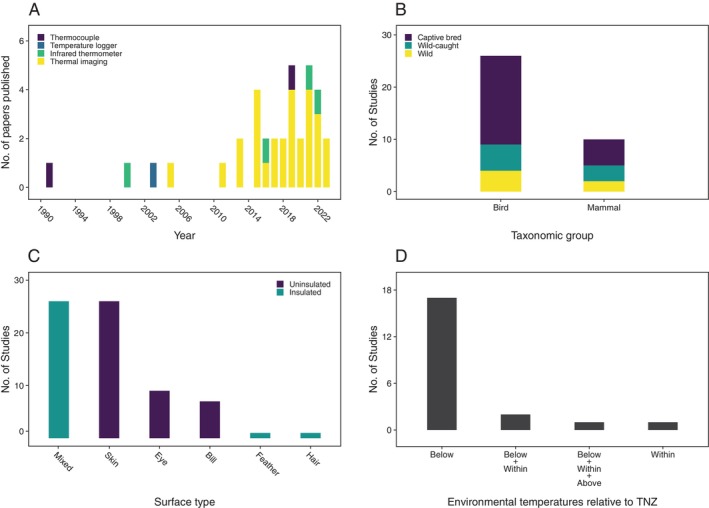
Numbers of included papers/studies (see Section II.3) investigating relationships between body surface temperatures and stress by (A) body surface temperature measurement method, grouped by year of publication, (B) subject population type, grouped by taxon, (C) insulation level of surface from which temperatures were measured, grouped by surface type and (D) relationship between environmental temperatures experienced during data collection and subject thermoneutral zone (TNZ) for species where such data were available. Thermoneutral zone categories separated by plus signs indicate mixed study conditions (e.g. Below+Within means conditions were both below and within the thermoneutral zone).

**Fig. 4:**
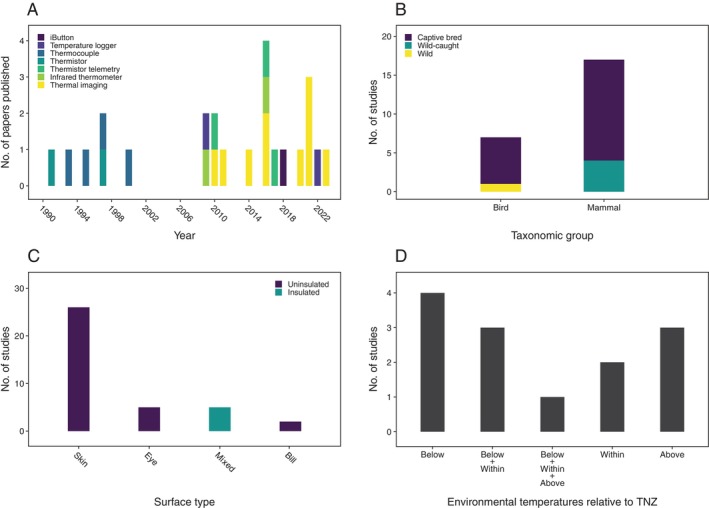
Numbers of included papers/studies (see Section II.3) investigating relationships between body surface temperatures and immune responses by (A) body surface temperature measurement method, grouped by year of publication, (B) subject population type, grouped by taxon, (C) insulation level of surface from which temperatures were measured, grouped by surface type and (D) relationship between environmental temperatures experienced during data collection and subject thermoneutral zone (TNZ) for species where such data were available. Thermoneutral zone categories separated by plus signs indicate mixed study conditions (e.g. Below+Within means conditions were both below and within the thermoneutral zone).

Publication of papers relating body surface temperatures to all four physiological functions has increased markedly since around 2010 (Figs 1A, 2A, 3A and 4A). Investigations of links between body surface temperatures and thermoregulation were the most frequently published (≥3 papers published per year since 2015), followed by those investigating stress (≥2 papers per year since 2015). By contrast, publication rates for immune response, and particularly metabolism remain relatively low with only one paper per year published in each functional group in most years since 2015.

Publication rate growth in recent years appears to have resulted from the opportunities associated with increased thermal imaging camera availability, as most papers published since 2010 used thermal imaging to assess body surface temperatures (Figs 1A, 2A, 3A and 4A). In terms of relative publication rates, the high frequency of thermoregulation papers presumably reflects widespread efforts to understand abilities of endothermic taxa to adapt to a warming climate (Cunningham, Gardner & Martin, 2021). However, the limited interest in linking body surface temperatures with metabolism or immune activity appears to represent a missed opportunity given the considerable potential utility of non‐invasive field methods to infer these functions. As such, greater targeting of the less‐understood links between surface temperature and these functions would be of value in broadening the range of physiological processes that might be inferred *via* body surface temperatures.

### (3) Thermoregulation

#### (a) Subjects

An approximately equal proportion of studies investigated birds and mammals (Fig. 1B), with most being captive bred (72% of studies), although only 16% of species studied were domesticated. Also, more than one and a half times more studies targeted adults (*N* = 44) than juveniles (*N* = 27) (subject life stage was not reported in four studies). If species number can be considered a reasonable proxy for relative research importance, then the near‐equal proportions of studies targeting mammals and birds suggests a potentially inappropriate bias towards the former. Estimates suggest there are around three times more bird species than mammal species (Barrowclough *et al*., 2016; Burgin *et al*., 2018). Furthermore, selective pressures in captivity are distinct from those experienced in nature (Frankham, 2008), and this can generate genetic and phenotypic divergence between captive and wild populations (Johnsson, Höjesjö & Fleming, 2011; Fraser *et al*., 2019). Consequently, allocating greater effort not only towards birds, but also towards more wild species from both endotherm groups would be desirable in future thermoregulation‐related research. Because only a minority of individuals survive to maturity in many species, increasing a focus on juveniles would substantially improve overall understanding, not least in terms of thermoregulatory development.

#### (b) Methods used

While most papers used an experimental approach (62%), a relatively large proportion (38%) were observational (Table 5A). Experimental evidence is generally considered more reliable (Boon *et al*., 2022), suggesting that a greater future emphasis on experimental designs would be valuable. Within the experimental group, approximately equal numbers of studies exposed subjects to acute and chronic challenges (Table 5A). However, natural thermoregulatory challenges are more likely to be chronic (defined here as ≥4 h) than acute (≤4 h), suggesting that more use of chronic challenges would make stronger connections with natural conditions. Most studies (97%) varied surrounding air/water temperatures as the challenge, with the remainder using conductive surfaces to cool or heat specific body regions. Skin was the most frequently targeted surface (Fig. 1C), with an even split between insulated and non‐insulated surfaces (both *N* = 87 studies).

**Table 5:** Numbers of included studies investigating relationships between body surface temperatures and (A) thermoregulation, (B) metabolism, (C) stress and (D) immune response, with the number of papers from which these studies were split in parentheses (see Section II.3). Studies were categorised by methodological approach, challenge exposure type, whether sex differences were analysed, whether a physiological validation was attempted, and the potential for study methods to have induced a confounding thermal physiological stress response.

Attribute	Total studies	Categorisation					
**(A) Thermoregulation**							
Approach	68 (68)	Observational		26 (26)		Experimental	42 (42)
Exposure type (experimental studies only)	43 (42)	Acute		23 (23)		Chronic	20 (20)
Sex differences analysed	69 (68)	Yes		10 (10)		No	59 (58)
Physiological validation attempted	68 (68)	Yes		40 (40)		No	28 (28)
Method stress potential	68 (68)	High	26 (26)	Low	20 (20)	Unclear	22 (22)
**(B) Metabolism**							
Approach	14 (14)	Observational		7 (7)		Experimental	7 (7)
Exposure type (experimental studies only)	7 (7)	Acute		2 (2)		Chronic	5 (5)
Sex differences analysed	14 (14)	Yes		2 (2)		No	12 (12)
Physiological validation attempted	14 (14)	Yes		5 (5)		No	9 (9)
Method stress potential	15 (14)	High	4 (4)	Low	9 (8)	Unclear	2 (2)
**(C) Stress**							
Approach	35 (35)	Observational		2 (2)		Experimental	33 (33)
Exposure type (experimental studies only)	34 (33)	Acute		32 (32)		Chronic	2 (2)
Sex differences analysed	35 (35)	Yes		6 (6)		No	29 (29)
Physiological validation attempted	35 (35)	Yes		11 (11)		No	24 (24)
Method stress potential	35 (35)	High	12 (12)	Low	20 (20)	Unclear	3 (3)
**(D) Immune response**							
Approach	24 (24)	Observational		1 (1)		Experimental	23 (23)
Exposure type (experimental studies only)	23 (23)	Acute		19 (19)		Chronic	4 (4)
Sex differences analysed	24 (24)	Yes		0		No	24 (24)
Physiological validation attempted	24 (24)	Yes		18 (18)		No	6 (6)
Method stress potential	24 (24)	High	11 (11)	Low	10 (10)	Unclear	3 (3)

Sex differences were investigated infrequently (<15%; Table 5A), which may constitute a serious weakness in the literature (see Section III.3.*c*). Only 59% of papers attempted a validation of thermoregulatory activity (Table 5A), for example by measuring internal body temperatures, indicating that overall rigour could be improved. Additionally, only one‐third of papers used methods considered as having unambiguously low potential for inducing a confounding thermal stress response, such as those discussed in Section III.5.*c* (Table 5A). This highlights that ‘low stress’ non‐invasive measurement methods should probably be prioritised more frequently (although see Section III.3.*c*). Lastly, within studies investigating species where thermoneutral zone data were available (*N* = 44), most exposed subjects to environmental temperatures combining conditions below, within and above, or below and within their thermoneutral zone (Fig. 1D).

#### (c) Outcomes

**Fig. 5:**
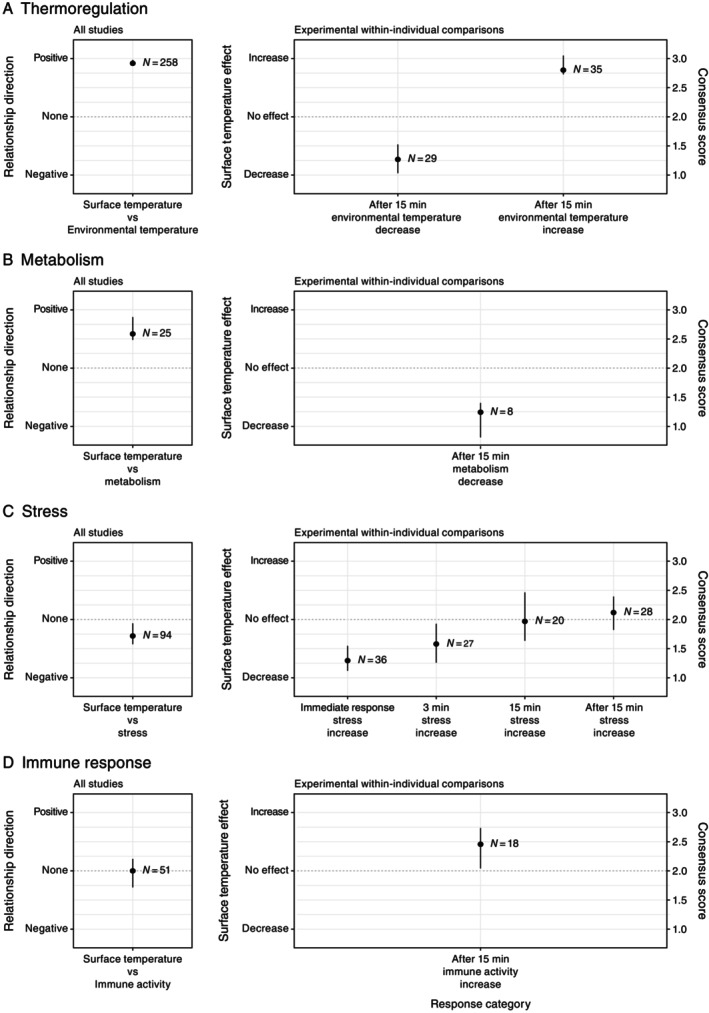
Body surface temperature *versus* challenge relationships across all included studies (observational and experimental), and challenge effects on body surface temperatures from included experimental within‐individual comparisons for research investigating surface temperature effects of (A) thermoregulation, (B) metabolism, (C) stress and (D) immune activity. Outcomes (positive, negative or no relationship, and increase, decrease, or no effect on surface temperature are derived from consensus scores calculated for each study (see Section II.4). Error bars are 95% confidence intervals calculated from the mean response.

The 68 papers on thermoregulation were split into 276 studies (see Section II.3). However, we excluded 18 studies (from two papers) as they did not provide true sample sizes, only numbers of thermal images. The overall consensus score for the predominant reported body surface *versus* environmental temperature relationship was strongly positive (Fig. 5A). Unequivocal and primarily positive relationships between body surface and environmental temperatures were reported in 95% of studies (Table 6A), whereas negative relationships were observed in 1% of studies, and 4% found no relationship.

**Table 6:** Numbers of included studies, with the number of papers from which studies were split in parentheses (see Section II.3), categorised by functional group (A, E – thermoregulation; B, F – metabolism; C, G – stress, D, H – immune response) and outcome. Outcomes (no relationship, negative or positive) for all studies refer to the predominant relationship reported between comparator and body surface temperature. Outcomes (above, below or no difference) for experimental within‐individual comparisons refer to body surface temperature difference from baseline condition. ‘Unequivocal’ outcomes were only e.g. positive, whereas variable outcomes which were mainly e.g. positive are listed as ‘Primarily’.

All studies (observational and experimental)	Total studies	No relationship			Negative			Positive		
		Unequivocal	Primarily	Total	Unequivocal	Primarily	Total	Unequivocal	Primarily	Total
**(A) Thermoregulation**										
Predominant relationship	258 (66)	10 (5)	–	10 (5)	3 (2)	–	3 (2)	239 (64)	6 (5)	245 (66)
**(B) Metabolism**										
Predominant relationship	25 (14)	8 (4)	–	8 (4)	–	–	–	17 (13)	–	17 (13)
**(C) Stress**										
Predominant relationship	94 (35)	17 (13)	–	17 (13)	47 (26)	3 (1)	50 (26)	27 (13)	–	27 (13)
**(D) Immune response**										
Predominant relationship	51 (24)	13 (10)	–	13 (10)	20 (13)	–	20 (13)	18 (11)	–	18 (11)

Experimental within‐individual comparisons	Total studies	No difference			Below			Above		
		Unequivocal	Primarily	Total	Unequivocal	Primarily	Total	Unequivocal	Primarily	Total
**(E) Thermoregulation**										
After 15 min (environmental temp. decrease)	29 (14)	2 (2)	–	2 (2)	22 (12)	2 (2)	24 (13)	3 (2)	–	3 (2)
After 15 min (environmental temp. increase)	35 (19)	–	–	–	2 (2)	–	2 (2)	31 (17)	2 (2)	33 (19)
**(F) Metabolism**
After 15 min (metabolism decrease)	8 (4)	1 (1)	–	1 (1)	7 (4)	–	7 (4)	–	–	–
**(G) Stress**
Immediate response (stress increase)	36 (14)	6 (4)	–	6 (4)	24 (13)	3 (1)	27 (13)	3 (2)	–	3 (2)
3 min (stress increase)	27 (10)	4 (3)	–	4 (3)	14 (8)	3 (1)	17 (8)	6 (3)	–	6 (3)
15 min (stress increase)	20 (6)	5 (3)	–	5 (3)	4 (2)	3 (1)	7 (3)	8 (5)	–	8 (5)
After 15 min (stress increase)	28 (11)	13 (7)	–	13 (7)	3 (3)	3 (3)	6 (4)	9 (6)	–	9 (6)
**(H) Immune response**
After 15 min (immune activity increase)	18 (10)	7 (5)	–	7 (5)	2 (2)	–	2 (2)	9 (7)	–	9 (7)

The overall consensus score was supported by experimental studies making within‐individual comparisons for responses after 15 min to both decreasing and increasing environmental temperatures (decreasing *N* = 29, increasing *N* = 35, Table 6E, Fig. 5A). The consensus score for responses to decreasing environmental temperatures indicated decreasing body surface temperatures (83% of studies reported decreases, 10% increases, and 7% found no response), whereas the consensus score for responses to increasing environmental temperatures indicated increasing body surface temperatures (94% of studies reported increases, 6% decreases, with none failing to respond). Consequently, the experimental literature indicates a robust body surface temperature change matching the direction of environmental temperature change at least 15 min after challenge onset.

**Fig. 6:**
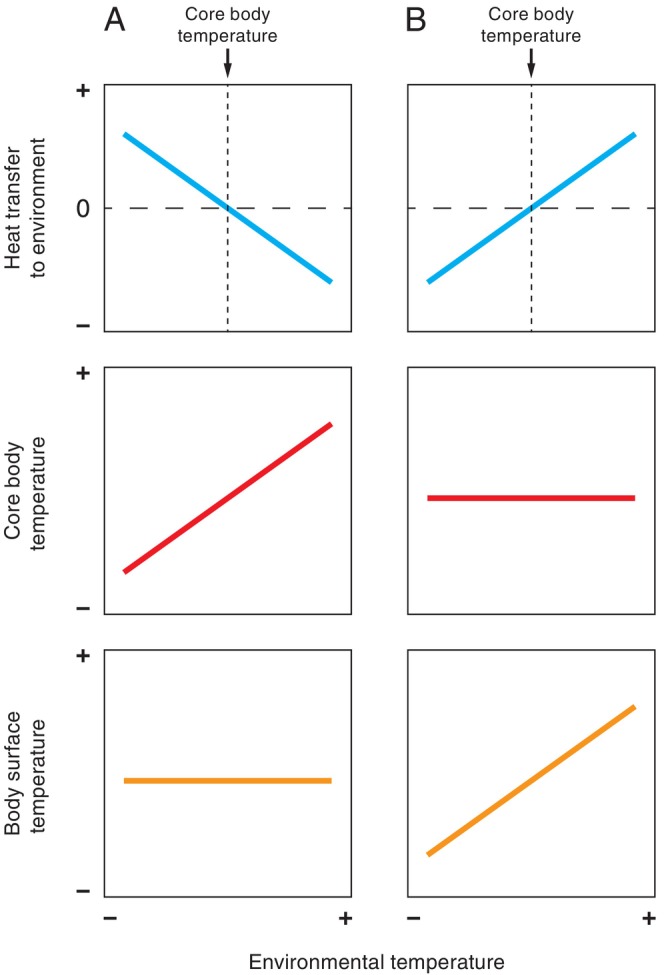
Schematics of body temperature and heat loss changes in relation to environmental temperature, where metabolic heat production is stable, and when either (A) body surface temperature or (B) core body temperature are held constant.

The strong positive consensus relationships are unsurprising. All body temperatures reflect the interplay between heat produced and heat lost; any regional body temperature will remain constant only when heat production equals heat loss. Homeothermic endotherms maintain a high, and relatively constant internal body temperature through high metabolic heat production (Clarke, 2017). Consequently, if metabolic rate and body surface temperatures were to be kept constant as environmental temperature increases (Fig. 6A), internal body temperatures would necessarily rise. This is because, in accordance with the second law of thermodynamics, heat transfer rate decreases with a lower temperature gradient (Atkins, 2010). Therefore, in situations where environmental temperature increases while surface temperatures are held stable, less of the metabolic heat generated at the body core can be transferred from the body surface to the environment (Lovegrove, Heldmaier & Ruf, 1991). Additionally, once environmental temperatures become higher than body surface temperatures, heat is transferred down the temperature gradient from the environment to the body. Conversely, maintaining a relatively constant internal body temperature with increasing environmental temperature (Fig. 6B) requires body surface temperatures to change with increasing environmental temperatures. Broadly speaking (and again assuming a relatively constant metabolic rate), at lower environmental temperatures, body surface temperatures must decrease to reduce heat loss to the environment, whereas at higher environmental temperatures, body surface temperatures must increase to elevate heat loss to the environment.

Heat flow to and from the environment is orchestrated by the hypothalamus, which integrates information from temperature‐sensing proteins [transient receptor channel proteins (TRPs)] situated at the body core, on mucous membranes and within the skin surface (Clarke, 2017). TRP output is processed by the posterior hypothalamus, which mediates physiological and behavioural thermoregulatory responses *via* additional hypothalamic, autonomic and cerebral thermoregulatory centres. Physiological thermoregulatory effectors at or near the body surface are predominantly autonomically driven, and either generate, retain or dissipate heat (Tan & Knight, 2018). Surface (or near‐surface) heat‐generating mechanisms comprise brown adipose tissue (BAT) thermogenesis (mammals only), and skeletal muscle shivering, whereas heat can be retained in the body core by vasoconstriction at the body surface (reducing the flow of warm blood from core to surface where heat can be radiated away to the environment), potentially combined with pilo/ptilo‐erection (increasing the depth of fur/feather insulation). By contrast, vasodilation is used to dissipate heat, along with evaporation [either through sweat (mammals only), panting, or saliva spreading], also potentially combined with a reduction in pilo/ptilo‐erection (Clarke, 2017). It is likely all these processes contributed to the consensus relationships we report, given the heterogeneity of included studies. Nevertheless, determining exact contributions would be difficult as few studies were designed to isolate thermoregulatory mechanisms, and so necessary information is lacking. However, designing future experiments which take into account context such as environmental temperature change direction, and regional differences in surface temperature dynamics should allow relatively straightforward inference of the processes driving thermoregulatory body surface temperature changes.

Perhaps the chief implication of the consensus that surface temperatures almost always increase with environmental temperatures is that the slopes of body surface and environmental temperature relationships are likely to be more informative than the simple relationship direction. Increasing or decreasing slope values, for example, may indicate activation and/or de‐activation of thermoregulatory mechanisms or their physiological limits. For instance, van Vuuren, Kemp & McKechnie (2020) showed that southern ground hornbill (*Bucorvus leadbeateri*) beak surface temperatures matched air temperature (within 1–2 °C) between 10 and 20 °C, but increased rapidly between 20 and 22 °C. Beak temperatures around 6 °C higher than air temperature then tracked the slope of air temperature once again between 22 and 28 °C, above which the beak–air temperature gradient gradually decreased until the maximum measured air temperature (36 °C) was reached. This pattern clearly demonstrates the onset of active heat dumping from the beak surface between 20 and 22 °C, and most likely maximal vasodilation at 28 °C. In this sense, examining slopes of body surface and environmental temperature associations relative to thermoneutrality should also be highly informative (although thermoneutral zone data are currently too limited to apply this approach broadly in comparing groups/species).

Assessing relationship slopes is beyond the scope of this review. This is principally because interspecies comparisons will be strongly biased by effective surface area (the proportion of surface area involved in heat transfer) to volume ratios (Phillips & Heath, 1995). Estimations of these traits for the included studies would necessarily be inaccurate, not least as subject mass was rarely reported. Consequently, it would not be possible to account for the bias with confidence, meaning results would be unlikely to offer reliable information beyond that obtained from our quantitative consensus profile analysis. Nonetheless, future studies designed with the intention of deriving relationship slopes would be useful to fill this major gap. Despite this situation, our relationship direction data do at least allow us to distinguish between typical outcomes with a positive slope, and interesting and unusual examples where the slope was negative or zero. Negative relationships between body surface temperature and environmental temperature were reported for two species: the Damaraland mole rat *Fukomys damarensis* (McGowan *et al*., 2020) and the domestic pigeon *Columba livia* (Østnes & Bech, 1998). Damaraland mole rats were found to reduce skin surface temperature during sequential exposure to increasing incubator air temperatures (from 12 °C to 33 °C). This response may relate to their somewhat extreme adaptations for conserving water and energy, including an unusually wide thermoneutral zone and low resting metabolic rate (Lovegrove, 1986; Bennett, Clarke & Jarvis, 1992; McGowan *et al*., 2020). In the pigeons, increased breast surface temperatures during cooling within a respirometry chamber (from 28 °C to −10 °C) most likely resulted from elevated metabolic heat production (potentially through shivering thermogenesis) in the pectoral muscles (Østnes & Bech, 1998). It is important to note, however, that in both cases, ‘positive slope’ surface–environment temperature relationships were reported from different body regions in the same papers. As such, regional functional roles (thermoregulatory or otherwise) are also likely to contribute to contrasting outcomes, making informed measurement site selection vital.

By contrast, 10 studies (from five papers) reported no relationship between surface and environmental temperatures (i.e. where the relationship is assumed to have a ‘zero slope’). Strikingly, all five papers studied birds, with four out of the five targeting domesticated species [chicken *Gallus gallus domesticus* (Brummermann & Reinertsen, 1992; Chang *et al*., 2018; Aswathi *et al*., 2019); Japanese quail *Coturnix coturnix japonica* (Santos *et al*., 2019)], and the remaining paper investigating multiple captive hummingbird species (Powers *et al*., 2017). Two of the chicken papers exposed birds to similar air temperatures expected to induce heat stress [Chang *et al*. (2018) = 36 °C; Aswathi *et al*. (2019) = 37 °C], while the third used an aluminium thermode to expose thorax skin to temperatures from 35 to 15 °C in 5 °C increments (Brummermann & Reinertsen, 1992). The Japanese quail paper contrasted surface temperature responses to environmental temperatures within (23 °C) and below (17 °C) “thermal comfort” (Santos *et al*., 2019), and the hummingbird paper exposed hovering individuals to temperatures between 18 and 30 °C (Powers *et al*., 2017). Given the relatively heterogenous methods used in these studies, it is tempting to conclude their examples of ‘zero slope’ relationships are more likely to relate to the group (i.e. birds), or domestication of the species investigated. Nevertheless, as with the ‘negative slope’ relationships described above, all papers reporting ‘zero slope’ relationships also reported ‘positive slope’ relationships from other body regions. Therefore, contrasting region‐specific functions are also likely to play a role in these unusual responses.

Six studies (from five papers) also reported inconsistent (albeit predominantly positive) within‐body‐region relationships between body surface temperatures and environmental temperatures. Relationships varied either across temperature ranges [e.g. positive relationship between body surface and environmental temperatures above environmental temperatures of 10 °C only (Doncaster *et al*., 1990); positive relationship between back surface and environmental temperatures above 24 °C only (Chang *et al*., 2018)], season [e.g. positive relationship between tarsus surface and environmental temperatures except in late winter; Nord & Folkow, 2017)], or time of day [(e.g. positive relationship between face surface and environmental temperatures only in morning; Kim *et al*., 2021b)]. As such, variation in the body surface–environmental temperature relationship might reflect changes in the relative importance of internal and surface thermoregulatory processes (and potentially behaviours) associated with crossing thermoneutral zone boundaries (Clarke, 2017), or seasonally increased thermogenesis (Nord & Folkow, 2017).

Despite the clear theoretical expectation for a strong positive consensus among body surface *versus* environmental temperature relationships, it remains notable that so few exceptions occurred across such a heterogenous range of species, body masses and habitats. For example, average bird body mass ranged from the 2.6 g calliope hummingbird (*Stellula calliope*) – the smallest native bird in North America (Powers *et al*., 2017) – to the 30–60 kg cassowary (*Casuarius casuarius*) (Eastick *et al*., 2019), whereas the mammal average mass range was even greater – from the ~9 g brown long‐eared bat (*Plecotus auritus*) (Webb, Speakman & Racey, 1993) to the 3000+ kg African elephant (*Loxodonta africana*), the largest living terrestrial mammal (Weissenböck *et al*., 2010). Similarly, while all bird subjects were terrestrial and live above‐ground, mammal subjects also included fossorial species such as the star‐nosed mole (*Condylura cristata*) (Tattersall & Campbell, 2023) and mole rats (McGowan *et al*., 2020), along with marine species including northern elephant seals (*Mirounga angustirostris*) (Norris, Houser & Crocker, 2010; Codde *et al*., 2016), harbor seals (*Phoca vitulina*) (Hansen, Lavigne & Innes, 1995) and bottlenose dolphins (*Tursiops truncatus*) (Noren *et al*., 1999). Clearly, almost all homeothermic endotherms operate within similar biophysical boundaries, despite substantial variation in size, habitat and lifestyle.

Interestingly, whether a thermoregulatory challenge was validated by change in internal temperatures (*N* = 72), or not (*N* = 186) did not appear to affect the proportions of positive/negative predominant reported body surface *versus* environmental temperature relationships (Table S1A). Similarly, surface temperature responses after 15 min in the experimental within‐individual comparison subset also did not differ with validation status (increase: validated *N* = 21, unvalidated: *N* = 14, decrease: validated *N* = 16, unvalidated: *N* = 13, Table S1A). Only three of the 72 validated studies found negative relationships between environmental temperature and internal temperature (increasing internal body temperature with decreasing environmental temperature). The two papers containing these studies both exposed subjects to somewhat extreme cold challenges [Østnes & Bech (1998) minimum −10 °C; Nord & Folkow (2017) minimum −30 °C]. Conversely, all 68 validated studies reporting unequivocally positive relationships exposed subjects to temperatures above 0 °C. Therefore, these negative internal body *versus* environmental temperature relationships most likely relate to strong metabolic rate increases made to cope with extreme cold challenge/heat loss. This assertion is supported by the negative relationships between metabolic rate and environmental temperature reported by Nord & Folkow (2017), and between pigeon breast surface temperature and environmental temperature reported by Østnes & Bech (1998).

Only 35 studies from 10 papers investigated sex differences, with nine studies (from five papers) reporting sex effects. However, even those outcomes were mixed. For example, two mammal (domestic rabbit and cattle) and one bird (pied babbler *Turdoides bicolor*) paper reported higher body surface temperatures in males than in females (Thwaites, Baillie & Kasa, 1990; Abduch *et al*., 2022; Soravia, Ashton & Ridley, 2022). However, Soravia *et al*. (2022) found male pied babblers only to have higher body surface temperatures below environmental temperatures of 38 °C. And, a further study on rabbits reported higher body surface temperatures in females, albeit in only one of three trials (de Lima *et al*., 2013). Most conclusively, high‐temperature incubation eliminated adult bill surface temperature responses to increased environmental temperature in female Japanese quails that were observed in birds of both sexes when incubated at lower temperatures (Carvalho *et al*., 2020). So, while reported sex effects were relatively minor, they did occur in approximately 25% of studies where they were examined, and have the potential to affect results. Therefore, the literature suggests that future studies should routinely assess sex differences.

Importantly, there were no discernible impacts of whether the methods used had the potential to induce a confounding thermal stress response on outcomes (Table S2A). This suggests that thermoregulatory surface temperature effects outweigh those related to stress. Therefore, while it would seem a sensible precaution to prioritise non‐invasive body surface temperature measurement techniques in this context, not doing so is unlikely to be a major issue.

### (4) Metabolism

#### (a) Subjects

An approximately equal proportion of metabolism studies investigated birds and mammals (Fig. 2B), although there were only 14 studies overall. However, most studies examining birds (55%) used captive animals, whereas most investigating mammals targeted wild species (56%; Fig. 2B). Domestic animals comprised 33% of species, which were used as subjects in 27% of studies. More than twice as many studies targeted adults (*N* = 9) than juveniles (*N* = 4), with one including both life stages within the same analysis (a comparison of surface temperatures with moult stage in juvenile and adult elephant seals *Mirounga leonina*; Paterson *et al*., 2022). As for the thermoregulation studies, equal proportions targeting mammals and birds suggests a potentially inappropriate bias towards the former (assuming that species numbers are considered a reasonable proxy for relative research importance). Consequently, greater future emphasis on studying birds, particularly wild species, would broaden understanding of metabolism–surface temperature relationships in natural environments. Increased targeting of juveniles across both birds and mammals would also likely be beneficial in terms of providing more complete insights across life stages.

#### (b) Methods used

All studies assessed body surface temperature responses to metabolic challenges (e.g. before and during a given challenge), rather than attempting to compare surface temperatures and metabolism at explicitly defined states (e.g. basal, resting, summit or maximum metabolic rate). While the former is certainly more practical in most situations (and likely reflects natural circumstances better), pursuing the latter strategy in future research may also be useful in characterising relationship limits.

There were equal numbers of experimental and observational papers (Table 5B). Within the experimental group, more than twice as many papers exposed subjects to chronic metabolic challenges than acute (Table 5B). Acute metabolic challenges (e.g. prevention of foraging due to weather conditions, predator or social competitor presence) are likely to be experienced most often by wild animals, with chronic challenges (e.g. courtship, breeding, moult) often occurring only annually. Therefore, assessing surface temperature effects of acute (<4 h) metabolic challenges more frequently in future research should better characterise the full lived experience of free‐living species.

Experimental papers used food restriction as the metabolic challenge (*N* = 7), while observational papers examined correlations between body condition (*N* = 3) or moult (*N* = 4) and body surface temperatures. It is noteworthy that experimental within‐individual comparisons only examined surface temperature responses to decreased metabolism. Consequently, there is a clear need for experimental investigations that increase metabolic challenges (e.g. by inducing moult). Only one paper measured surface temperature responses after a metabolic challenge had ceased (Winder *et al*., 2020). More frequent study of surface temperature responses during recovery from metabolic challenge could be especially useful in terms of providing new tools for understanding resilience.

Hair and mixed surfaces were the most frequently investigated (Fig. 2C), meaning most were insulated to some extent. Over one‐third of studies targeting insulated surfaces found no relationship between metabolic activity and body surface temperature, whereas all but one of nine studies targeting uninsulated areas observed a positive relationship. Body temperature is generally expected to track variation in metabolic rate (e.g. Refinetti, 2020). So, at least when changes in heat loss relating to environmental conditions are accounted for [as they were in two papers targeting insulated surfaces that failed to find relationships (Walcott, Kirkham & Burns, 2020; Guerrero, Rogers & Sepúlveda, 2021)], body surface temperature should have a positive relationship with underlying metabolic activity. As insulated body surface temperatures are buffered from changes in heat loss at the skin surface, the lack of reported relationships between insulated surface temperatures and metabolic activity likely results from this buffering. Accordingly, future studies should focus on uninsulated surfaces where possible.

Sex differences were again investigated infrequently (<15%; Table 5B). Also, few papers (36%; Table 5B) attempted to validate metabolic activity, and almost half (40%) used methods with high or unclear potential to induce a confounding thermal stress response. Just as importantly, where subject thermoneutral zone data were available (*N* = 9), no studies were conducted at environmental temperatures above the thermoneutral zone (Fig. 2D). Given likely interactions between thermoregulatory activity and metabolism, body surface temperature–metabolic activity relationships should be investigated across the widest range of conditions possible to ensure outcomes are understood across all thermoregulatory states.

#### (c) Outcomes

The 14 papers were split into 25 studies (see Section II.3). The overall consensus score for the predominant body surface temperature *versus* metabolic activity relationship reported was broadly positive (Fig. 5B). Unequivocally positive relationships between body surface temperatures and metabolic activity were reported in 68% of studies, whereas 32% found no relationship. A negative body surface temperature *versus* metabolic activity relationship was not observed in any study. The overall consensus score was supported by experimental studies making within‐individual comparisons for responses after 15 min to decreasing metabolic activity (*N* = 8, Table 6F, Fig. 5B). The consensus score for responses to decreasing metabolism indicated decreasing body surface temperatures 15 min or more after challenge onset (88% of studies reported decreases, while none observed an increase, and 12% found no response).

**Fig. 7:**
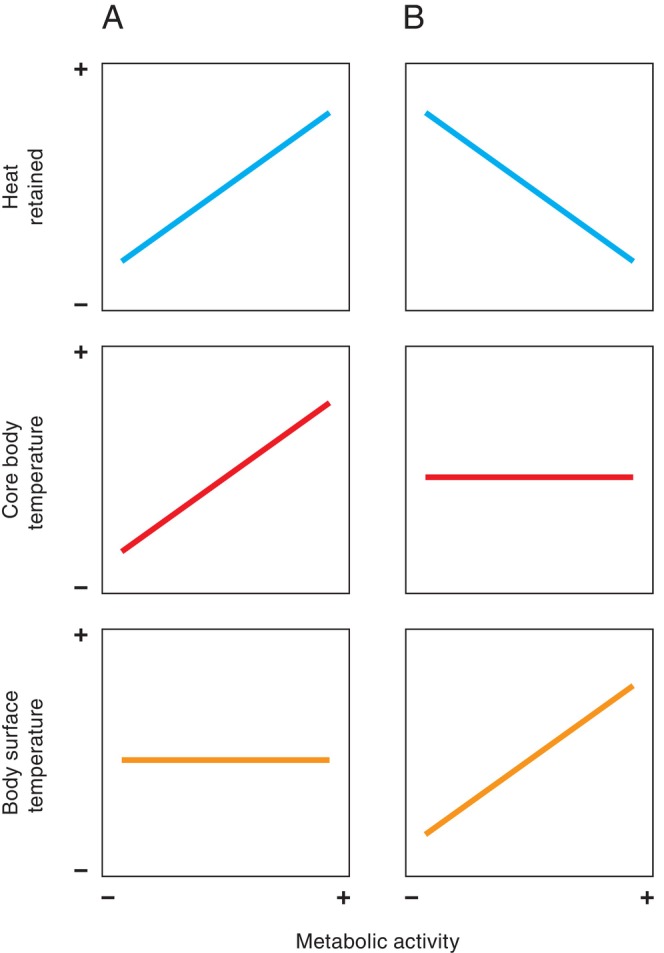
Schematics of body temperature and heat retention changes in relation to metabolic activity (i.e. heat production), where environmental temperature is constant, and when either (A) body surface temperature or (B) core body temperature are held constant.

The broadly positive relationship between body surface temperatures and metabolic activity across most studies confirms the expected association that body surface temperatures should generally track metabolic rate (Refinetti, 2020). As discussed above (in Section III.3.*c*), regional body temperatures can remain constant only when heat production equals heat loss. Heat production is an inevitable result of metabolic activity, as only some of the energy released by oxidation can be recovered in chemical form (Ricquier, 2006). Therefore (assuming a stable environmental temperature) maintenance of a constant surface temperature with increasing metabolic activity must increase heat retention, because heat loss at the body surface cannot rise with heat production (Fig. 7A). Clearly then, the retained heat would necessarily increase core body temperature as metabolic activity increases. Consequently, for homeothermic endotherms to maintain their relatively constant core body temperature (Clarke, 2017) in a stable environmental temperature, surface temperatures must increase with metabolic activity, to allow transfer of excess heat to the environment (Fig. 7B).

It follows then that the mechanisms driving the largely positive body surface *versus* metabolic activity relationship consensus are most likely the same autonomically mediated surface (and near‐surface) effectors described in Section III.3.*c*. Indeed, indicators of change in metabolic activity detectable at the body surface are essentially thermoregulatory in nature. In this sense, metabolic and thermoregulatory body surface temperature biomarkers are likely integrated in ways that may be difficult to parse in some circumstances. For example, heat generated by mitochondrial metabolism or muscular activity in excess of that needed to maintain core body temperature will be transferred to the environment *via* vasodilation, evaporation or reductions in pilo/ptilo‐erection. In such situations, inferring metabolic changes through surface temperature dynamics should be relatively straightforward. However, during environmental temperature reduction, metabolic heat production could conceivably be increased to maintain a stable core temperature without any excess needing to be dumped to the environment. Similarly, the reverse may occur where metabolic rate is reduced in response to increasing environmental temperatures.

This situation has a number of ramifications for those wishing to infer metabolism from surface temperatures. Firstly, given temperature biomarkers of metabolic rate change at the body surface are thermoregulatory, it will be essential to account for environmental temperatures to be able to determine metabolic functions beyond changes made in response to environmental temperature (e.g. changes dealing with food restriction, driving moult or powering activity). Second, including thermoneutral zone data in analyses – clearly demarcating those environmental temperatures where increased metabolic activity is necessary to maintain core temperatures – would undoubtedly assist in such function identification. Third, constant rapidly and substantially fluctuating environmental temperatures may prohibit detection of metabolic changes from body surface temperatures. Nonetheless, high‐temporal‐resolution environmental temperature monitoring should allow identification of the most stable periods likely to yield the best information.

Of the eight studies (from four papers) reporting no relationship, six (from two papers) investigated marine mammals (Walcott *et al*., 2020; Guerrero *et al*., 2021), and two investigated birds (Tattersall *et al*., 2016; Winder *et al*., 2020). As mentioned in Section III.4.*b*, the lack of expected relationships in the marine mammal studies likely results from insulating fur buffering skin temperature changes (although rapid changes in air temperature could also play a role). In the bird studies, Tattersall *et al*. (2016) did find a positive relationship between metabolic activity and body surface temperature in domestic ducks (*Anas platyrhynchos domesticus*) kept at thermoneutral temperatures, but not for individuals kept in cold conditions. The authors reasoned that thermoneutral birds used vasoconstriction at the bill surface to reduce heat loss during food restriction to save energy, while individuals kept at low temperatures could not, as vasoconstriction was already maximal. Consequently, surface temperature responses among the thermoneutral birds may have been only indirectly connected with reduced metabolism. Conversely, Winder *et al*. (2020) reported a positive relationship between metabolic activity and body surface temperature in the great tit (*Parus major*) bill, but not the eye region. Here, the authors hypothesised that eye region surface temperatures were held more constant than bill temperatures to maintain brain and/or optical function. Both bird cases highlight how regional thermoregulatory processes can both reveal (e.g. through close correlation between metabolic rate and surface temperature) or obscure (e.g. where physiological floor or ceiling effects limit the range over which metabolic rate and surface temperatures can correlate) links between body surface temperatures and metabolic activity. As such, it is vitally important to consider thermoregulatory functions of target surface regions as well as the context of environmental conditions when aiming to infer metabolic activity from body surface temperatures. In this sense, mechanistic studies determining the exact processes contributing to links between metabolism and body surface temperatures across a range of ambient conditions would be exceptionally valuable.

The only study to report body surface temperature responses after a metabolic challenge had ceased observed a rapid (almost immediate) return to baseline (Winder *et al*., 2020). Also, none of the five studies (from two papers) that investigated sex effects reported sex differences. Clearly, more data are required to determine if either pattern is typical. Validation of metabolic challenges appears important, given almost half (*N* = 7/16) of unvalidated studies (Table S1B) reported no relationship between metabolism and body surface temperatures, compared to only one of the nine validated studies. Contrastingly, responses after 15 min in the experimental within‐individual comparison subset did not appear to differ with validation status, notwithstanding the small sample size (validated: *N* = 5, unvalidated *N* = 3; Table S1B). It is noteworthy that only two of the nine validated studies used a ‘standard’ method for measuring metabolic rate to validate their procedures. Both Zhou & Yamamoto (1997) and MacLeod *et al*. (1993) used respirometry to assess O_2_ consumption (considered equivalent to metabolic rate; Green, 2011). The remaining seven studies used less‐commonplace (and more indirect) validation measures of energy expenditure, including body mass change (Ayala‐Berdon *et al*., 2017), rectal temperature (Mahjoubi *et al*., 2015), or energetic costs modelled from core body temperature changes (Tattersall *et al*., 2016). Therefore, it seems appropriate not only to recommend that challenges should routinely be validated when seeking to link metabolism and surface temperatures, but also that validation methods should be as direct as possible, to minimise potential for error.

It is conspicuous that across all studies, predominant relationships from those considered to use methods with low potential to produce a confounding thermal stress response (*N* = 16, Table S2B) were more equivocal than those using methods with high (*N* = 4), or unclear potential (*N* = 5) (see Table 2 for details of methods categorisation). However, this outcome appears coincidental. Given the reasoning outlined above, it seems most likely insulated target body surface regions explain why these studies found no relationship. Moreover, it is difficult to imagine a credible mechanistic reason linking low method stress potential and a lack of the expected relationship between metabolic activity and body surface temperatures. Also, responses after 15 min in the experimental within‐individual comparison subset did not appear to differ with method stress potential (low *N* = 2, high *N* = 2, unclear *N* = 4; Table S2B). Therefore, while thermal stress responses still might affect body surface temperature relationships with metabolic activity (and so caution is warranted in terms of method choices), the likelihood of this appears relatively low.

### (5) Stress

#### (a) Subjects

More than twice as many stress studies investigated birds than mammals, with most being captive bred (Fig. 3B). Domestic animals (mainly chickens) were the subject of 46% of studies, with 21% of species studied being domesticated. Four times as many studies targeted adults (*N* = 24) than juveniles (*N* = 6), with two studies including both life stages within the same analysis and three that did not report subject life stage. Assuming that species numbers are reasonable proxies for group relative research importance, birds are again underrepresented (albeit substantially less so than for thermoregulation, metabolism and immune studies). Nonetheless, a greater emphasis on both free‐living bird and mammal species in future work would improve understanding of stress‐induced surface temperature responses in the fluctuating conditions of wild habitats. Juvenile responses were the least investigated for stress responses among our four functional groups. This lack of information regarding development of the body surface temperature–stress response probably represents the largest knowledge gap relating to stress study subject choice.

#### (b) Methods used

Almost all included stress papers were experimental (Table 5C). Within the experimental group, almost all (93%) studies exposed subjects to acute challenges (Table 5C). Handling (*N* = 5) or capture and handling (*N* = 7) were the methods used most frequently to induce acute stress, although a considerable variety of alternatives were also employed (*N* = 24 other stressor types). While acute stress is the type most frequently experienced by wild animals, chronic activation of physiological stress systems may also be relatively common [e.g. from social conflict (Schoenle, Zimmer & Vitousek, 2018), predatory pressure (Boonstra, 2013) or human activity (Kaisin *et al*., 2021)]. Consequently, an almost exclusive focus on acute stress signifies a critical deficiency among existing work. Similarly, only one study explicitly investigated responses to reduced stress, and even then using an unnatural manipulation (using exogenous dexamethasone to downregulate HPA activity; Ouyang *et al*., 2021). Also, the few studies following acute stress responses from initiation through to ‘return to baseline’, were all conducted in captivity. Captivity is often stressful (Morgan & Tromborg, 2007), which likely affects recovery. Given that recovery is a key factor in resilience, and resilience is a core driver of fitness (Reed, Wolfe & Romero, 2024), it would potentially be extremely useful if it can be shown that natural acute and chronic stress recovery processes are reflected in body surface temperature dynamics.

Skin and mixed surfaces (*N* = 26 studies each), were most frequently targeted, with a majority of surfaces examined being uninsulated (Fig. 3C). However, the categories used in Fig. 3C likely underestimate the number of studies targeting uninsulated surfaces, as head, face and eye regions were classified as mixed surface, but were frequently measured *via* temperature maxima. This means the actual measurement probably came from an uninsulated region such as the medial canthus. As surface temperature changes during stress can be rapid (see Section III.5.*c*), avoiding insulated surfaces that buffer skin temperature dynamics would seem advisable.

Sex differences were once again investigated infrequently (17%; Table 5C), suggesting a likely deficit among stress studies (although see Section III.5.*c*). Also, only 31% of papers attempted to validate physiological stress (Table 5C). Most surprisingly given the subject matter, at least one‐third of papers used methods with high potential to induce a confounding thermal stress response when measuring baselines (Table 5C). Plainly, researchers investigating stress responses need most of all to avoid generating confounding stress effects through their choice of methods (see Section III.5.*c*). Finally, among studies where thermoneutral zone data were available, most exposed subjects to environmental temperatures below their thermoneutral zone (Fig. 3D). There is evidence that body surface temperature responses during stress are affected by environmental temperatures, and may even have a thermoregulatory function (Robertson, Mastromonaco & Burness, 2020). Hence, it seems especially important that the strong current bias towards measuring stress responses at temperatures below the subjects' thermoneutral zones is rectified to include all thermoregulatory states.

#### (c) Outcomes

The 35 papers were split into 94 studies (see Section II.3). As almost all studies (93%) investigated effects of acute stress, we assume our consensus outcomes effectively reflect only responses to acute stress, and discuss them as such below. The overall consensus score for the predominant body surface temperature *versus* acute stress relationship was negative, but only marginally (Fig. 5C). This indicates that when stress was applied, body surface temperatures tended to decrease. Unequivocally or primarily negative relationships between body surface temperatures and acute stress were reported in 53% of studies (Table 6C), whereas 29% observed positive relationships, and 18% found no relationship.

The overall consensus score appears likely to have been influenced, at least to some extent, by measurement timing. Consensus scores of experimental within‐individual comparisons for immediate responses (*N* = 36), and responses at 3 min (*N* = 27), 15 min (*N* = 20), and after 15 min (*N* = 28) to acute stress suggest a broadly negative immediate response, which dissipates by 15 min after stressor onset (Fig. 5C). Also, in a proportion of cases, the initial negative response appeared to be followed by a temporary rebound above baseline, although this effect was less generalised than the immediate response. The immediate response indicated decreasing body surface temperatures with increased stress (75% of studies reported body surface temperatures decreased, 8% increased, and 17% found no response; Table 6G). Similarly, at 3 min after stressor onset, body surface temperatures were below baseline in 63% of studies, above baseline in 22%, and no different from baseline in 15%. By 15 min, body surface temperatures were only below baseline in 35% of studies, above baseline in 40%, and no different from baseline in 25%. Finally, after 15 min, body surface temperatures were below baseline in 21% of studies, above baseline in 32%, and no different from baseline in 46%. Among studies with durations longer than 15 min where measurements continued post‐challenge, those reporting no return to baseline (15 studies from seven papers) ended measurements 1 h or less post stressor onset. The four studies (from three papers) in this group that observed a return to baseline before measurements halted found body surface temperatures recovered to baseline in less than 2.5 h.

The brief immediate decrease in body surface temperature during acute stress must be primarily under the control of the sympathetic–adrenal–medullary (SAM) system, as the hypothalamic–pituitary–adrenal (HPA) axis is much slower to respond (Sapolsky, 2002). And, while it was not directly measured in any included study, sympathetically mediated vasomotor activity appears the only plausible biological mechanism for such short‐term temperature changes at the body surface. However, the negative consensus score is already less clear at 3 min, and eradicated by 15 min after stressor onset. These later consensus response patterns probably result from multiple influences, potentially a composite of the discontinuation of vasomotor responses to acute stress, confounding physiological processes, interspecific response differences, and issues relating to lack of validation.

**Fig. 8:**
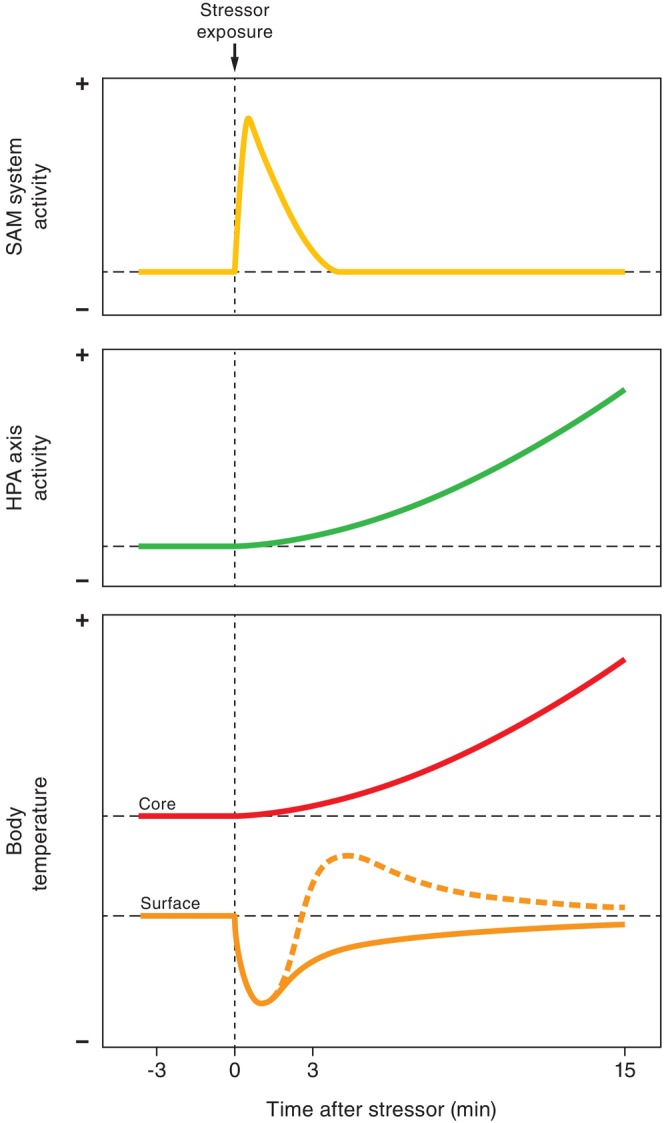
Schematics of body temperature, hypothalamic–pituitary–adrenal (HPA) axis activity, and sympathetic–adrenal–medullary (SAM) system activity responses to instantaneous acute physiological stress exposure. The surface temperature response pictured approximates that suggested by the results presented herein, with solid and dashed lines representing the apparent predominant and second most predominant patterns observed, respectively.

There are five main implications deriving from this analysis of the literature. First, the proportion of studies finding no difference from baseline was relatively low (≤17%) before 15 min, but increased to one quarter at 15 min, and again to almost half after 15 min (47% – although see discussion of potential validation effects below). This pattern follows what might be expected from ephemeral catecholamine‐driven (i.e. SAM system‐mediated) vasomotor activity. Second, while the rapid SAM response is likely to dissipate quickly (Sapolsky, 2002), it may continue beyond the point at which the HPA axis activates (typically 2–3 min post stressor onset; Romero & Reed, 2005). For example, increased heart rate, and reduced heart rate variability and bill temperatures persisted throughout 3 min of capture, handling and close human presence in house sparrows (*Passer domesticus*) (Jerem & Romero, 2023). Therefore, body surface temperature responses to stress after 2–3 min are likely (at least initially) due to a combination of SAM‐ and HPA‐driven processes (Fig. 8), which may act antagonistically [e.g. SAM‐mediated vasoconstriction reducing the flow of warm blood to the body surface (Jerem & Romero, 2023), while HPA‐mediated (non‐genomic) increases in energy mobilisation/metabolism (Lee *et al*., 2012) escalates heat flow to the body surface]. Equally, other less well‐understood mechanisms contributing to increased core body temperature in response to acute stress (stress‐induced hyperthermia; reviewed in Bouwknecht, Olivier & Paylor, 2007) such as the dorsomedial hypothalamus–medullary–sympathetic axis (Oka, 2018) could also play a role after the rapid initial SAM response. Third, while vertebrate stress anatomy is well conserved, interspecific regulatory differences in responses are likely (Romero & Gormally, 2019), which could also lead to opposing results, preventing a consensus response direction emerging. Fourth, it is clear from many papers that targeting different body regions resulted in contrasting responses. For example, all three studies that found immediate stress‐induced body surface temperature increases, and four of the six studies reporting no immediate response, were from papers where the opposite pattern was found in other body regions [Edgar *et al*., 2013a (head = increase, eye, comb = decrease); Campbell *et al*., 2019 (comb = decrease, eye region, head = no response); Nord & Folkow, 2019 (during winter at air temperature 20 °C, head = increase, back = decrease); Zuluaga & Danner, 2023 (eye, bill = decrease, eye region = no response)]. Similar situations occurred among all outcomes analysed, underlining again the importance of measurement site choice. Fifth, two discrepancies can result from failure to validate a physiological stress response. While interpretation is straightforward when an attempted validation reveals no stress response was generated (e.g. Iyasere *et al*., 2022) (i.e. no actual test of the relationship between body surface temperature and stress can be said to have taken place), not attempting validation at all makes drawing robust conclusions impossible. Across all outcomes, the proportion of studies reporting no effect of stress was markedly higher when studies did not attempt to validate a physiological stress response, e.g. *via* hormone analysis (Table S1C). This contrast was especially prominent within immediate surface temperature responses where all validated results indicated only reductions in surface temperature with stress [validated studies reported only decreases in surface temperature (*N* = 11), whereas unvalidated studies reported all three responses (increase *N* = 3, decrease *N* = 16, no response *N* = 6)]. The distribution of responses at 3 min with validated and unvalidated studies was similar (validated: decrease *N* = 8; unvalidated: increase *N* = 6, decrease *N* = 9, no response *N* = 4). Unfortunately, these data suggest that the results from unvalidated studies are unreliable since they may or may not have generated a stress response. Clearly, all five of the above factors should be taken into consideration when planning future stress studies.

The five studies where sex differences were found (50% of the studies where sex effects were investigated) came from two papers, and reported contrasting results. Where effects of rolling acute stressors on body surface temperature were greater in male black‐capped chickadees (*Poecile atricapillus*) (Robertson *et al*., 2020), responses to teasing were greater in female common marmosets (*Callithrix jacchus*) (Ermatinger, Brügger & Burkart, 2019). Robertson *et al*. (2020) hypothesised that differences in heat dissipation capacity existed between the sexes, or there were sex differences in selective pressure on thermoregulation. By contrast, Ermatinger *et al*. (2019) presumed the sex differences related to behavioural differences, where more‐aggressive female marmosets may experience higher magnitude responses. Nevertheless, regardless of the basis for this variation, between‐sex differences were relatively minor. Consequently, while precautionary analysis of sex effects in future work would be desirable given that so few studies have examined them, the limited current evidence suggests sex may not be a major factor in body surface temperature responses to stress.

Finally (and unsurprisingly), the potential for methods to induce a confounding thermal stress response does appear to require greater consideration in future studies. Reduced proportions of negative (i.e. decreasing) body surface temperature responses to stress (at 15 min, after 15 min, and for the predominant relationship reported across all observational and experimental studies) where methods with high stress potential were used suggests that at least in some cases, earlier response stages may have been missed (Table S2C).

### (6) Immune response

#### (a) Subjects

Over twice as many included immune studies investigated mammals as birds, with almost 80% of subjects in both taxonomic groups being captive bred (Fig. 4B), and 54% of species studied being domesticated. Also, one and a half times as many studies targeted adults (*N* = 9) than juveniles (*N* = 6), although more than one third of studies (*N* = 9) did not report subject life stage. Assuming (as above) global species numbers reflect relative research importance, birds are more underrepresented in immune studies than in those investigating thermoregulation, metabolism or stress. Similarly, immune studies have the greatest proportion of captive bred and domesticated subjects among our four functional groupings (Figs 1B, 2B, 3B and 4B). Accordingly, it would be beneficial for the purposes of inferring immune activity in wild animals to target birds as subjects, and more wild, or at least wild‐caught species in future work. Also, as with all other functional groupings, increased targeting of juveniles would be valuable in delivering a clearer picture of immune system effects on body surface temperatures across all life stages.

#### (b) Methods used

All but one paper was experimental (Table 5D), and 83% of experimental papers exposed subjects to acute (<4 h) immune challenges (Table 5D), with 62% of all studies using pyrogens and 38% using actual pathogens. This appears an appropriate initial strategy in that immune activity is probably more easily detected during acute challenges (Wobeser, 2007). Nonetheless, it would be equally useful to know whether chronic diseases with more mild and subtle effects can also be inferred from body surface temperatures. Therefore, more immune activity investigations on chronic timescales would be worthwhile. Additionally, no studies explicitly decreased immune activity, and all experimental within‐individual comparisons following immune responses through to recovery were conducted in captivity. Consequently, as for stress, it could prove immensely valuable if the entire immune response can be shown to be detectable from body surface temperature dynamics in natural environments.

Skin was the most frequently targeted surface, contributing to a high proportion of targeted surfaces being non‐insulated, and so likely more reliable data (Fig. 4C). However, sex differences were not examined in any immune paper (Table 5D), constituting a particularly stark knowledge gap. Most papers (75%) attempted a physiological validation of immune activity (Table 5D), indicating the highest rigour among our functional groupings, although still leaving room for improvement. Over half the papers were categorised as having used methods with high or unclear potential to induce a confounding thermal stress response (Table 5D), which may have influenced outcomes (see below). Within the 13 studies where thermoneutral zone data were available, subjects were exposed to environmental temperatures below, within and above the thermoneutral zone (Fig. 4D).

#### (c) Outcomes

The 24 papers were split into 51 studies (see Section II.3). The overall consensus score for the predominant body surface *versus* immune activity relationship reported was equivocal (Fig. 5D), highlighting minimal agreement between studies (35% reported positive relationships, 39% negative relationships, while 26% found no relationship; Table 6D).

The overall consensus score contrasted with experimental studies making within‐individual comparisons for responses after 15 min to increasing immune activity (*N* = 18, Table 6H, Fig. 5D). The consensus score for responses to increasing immune activity indicated increasing body surface temperatures 15 min or more after challenge onset (50% of studies reported increases, while 11% observed a decrease, and 39% found no response). Thirteen of the 18 studies reported that body surface temperature responses ceased beyond 15 min after an immune challenge with a mean ± SE response end time of 4.99 ± 1.18 h.

**Fig. 9:**
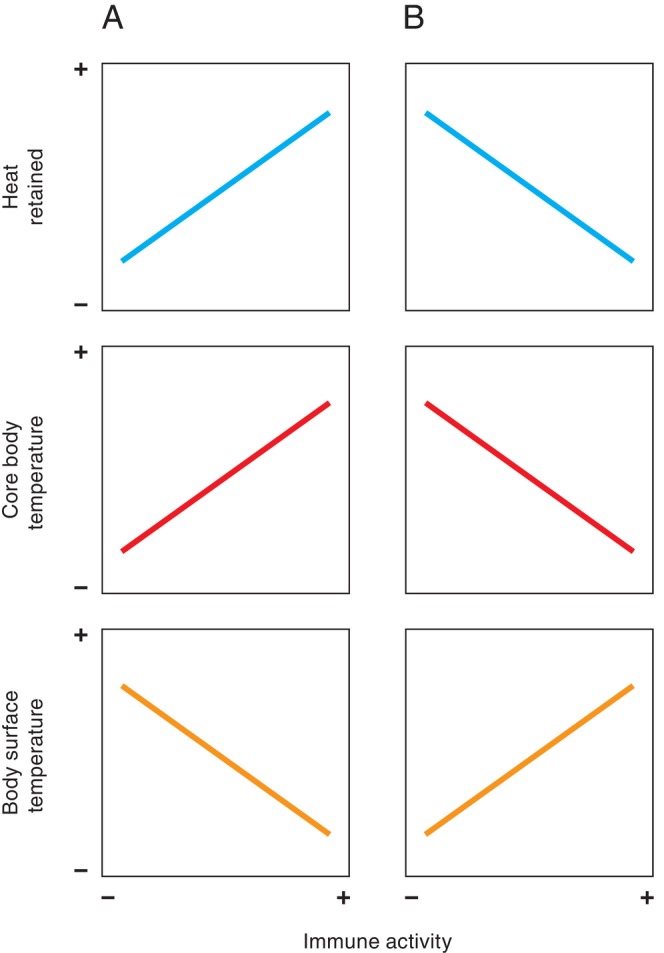
Schematics of body temperature and heat retention changes in relation to immune activity, where environmental temperature is constant, and either (A) within, or (B) below the thermoneutral zone (fever and regulated hypothermia, respectively).

Assuming that immune challenges generally result in processes involving increased body temperatures such as fever (Wright & Auwaerter, 2020) or inflammation (Antonelli & Kushner, 2017), the lack of a strongly directional overall consensus score may seem counterintuitive. However, a more complex picture emerges when considering the types of challenges and variety of environmental conditions experienced by subjects. For instance, 62% of immune studies used either lipopolysaccharides, interleukin 1ß or tumour necrosis factor α as their immune challenge. Although all are commonly referred to as ‘pyrogens’, each has been shown to induce either increased or decreased core body temperature depending on air temperature relative to the subject's thermoneutral zone (reviewed in Romanovsky, 2018). Typically, with these endotoxins core body temperature increases within thermoneutrality (fever; Fig. 9A), but is reduced when air temperatures are below thermoneutral (regulated hypothermia; Fig. 9B). Presumably then, air temperatures should also influence immune‐related peripheral vasomotor activity. At thermoneutrality, vasoconstriction to conserve heat (and so promote fever) would be expected (Evans *et al*., 2015), reducing body surface temperatures (Fig. 9A). Conversely, below thermoneutrality, vasodilation to dump heat to the environment (and so promote regulated hypothermia) would be expected, increasing body surface temperatures (Fig. 9B) (Romanovsky, 2004). Thermoneutral zone data was available for only 55% of immune study subject species. Even so, a range of environmental temperatures relative to thermoneutral were experienced within this group (Fig. 4D), and patterns of body surface temperature change reported among them support this hypothesis. Specifically, across all studies, positive immune challenge *versus* body surface temperature relationships were observed more than twice as often when experiments were carried out below (positive *N* = 5, negative *N* = 0, no relationship *N* = 3), rather than within (positive *N* = 1, negative *N* = 2, no relationship *N* = 2) or above (positive *N* = 2, negative *N* = 1, no relationship *N* = 1), the thermoneutral zone (Table S3). Similarly, body surface temperature increases after 15 min post immune challenge onset were four times as common below, rather than within the thermoneutral zone (Table S3). As such, it seems possible that rather than an overall consensus, immune challenges could induce varying body surface temperature responses, depending on environmental temperature.

Regardless, it is unlikely that environmental temperatures alone can account for all the reported variation in body surface temperature responses to immune challenge. Instead, additional factors are likely to have contributed. Firstly, inflammatory responses such as fevers can take place over multiple phases depending on challenge severity (Morimoto *et al*., 1988; Romanovsky *et al*., 1996; Romanovsky, Simons & Kulchitsky, 1998), between which body surface temperatures may differ markedly as vasomotor activity is used to manipulate core temperatures. Such effects could make measurement timing important, so that studies using different timetables will not be comparable. Also, both validation (or lack thereof) and contrasting potential for methods to induce a confounding thermal stress response appear strongly associated with variation in immune study outcomes. Proportionally, across all studies almost six times more validated studies (58%), reported negative relationships between immune activity and body surface temperatures than unvalidated studies (10%, Table S1D). On the other hand, method stress potential appears to have an even stronger effect. Fifteen times more negative than positive immune challenge *versus* body surface temperature relationships occurred in studies categorised as having high method stress potential (positive *N* = 1, negative *N* = 15, no relationship *N* = 4, Table S2D), with almost exactly the reverse pattern for studies that used methods with low stress potential (positive *N* = 16, negative *N* = 1, no relationship *N* = 5). In addition, 17 studies (from eight papers) occurred in both the validated and high‐stress‐potential groups, potentially linking specific validation methods (e.g. the invasive cloacal or rectal temperature measurements made in this overlapping group) with outcomes. To conclude, the immune studies data suggest that a strongly directional overall consensus profile among body surface temperature responses to immune challenge is unlikely, even if methodological issues are addressed in future work. Instead, it may be more useful to seek consensus profiles within specific thermoregulatory zones relative to thermoneutrality, and among studies with more regimented measurement timing schemes.

## IV. GENERAL DISCUSSION

In addition to the function‐specific points raised above, several more general issues arise from our analysis in the context of inferring physiological state from wild bird and mammal body surface temperatures. Firstly, we acknowledge that while splitting papers into individual ‘studies’ (see Section II.3) allowed us to describe accurately what data have been collected, a consequence of this methodology is that publications with many treatments and groups will have had a greater influence on our consensus calculations. Nevertheless, this effect is probably appropriate, as greater influence is provided to the most comprehensive research. More specifically, the most critical need we identified when characterising the current research landscape concerns establishing exactly which physiological mechanisms contribute to observed surface temperature dynamics. Mechanistic understanding of every relationship we report remains incomplete, and was rarely a focus of included studies beyond validating against a single physiological measure. Without more comprehensive insight, reliable interpretation of body surface temperatures will remain challenging. In this respect, combining detailed laboratory validations with confirmatory field studies would be invaluable.

Moreover, the ultimate aim of inferring physiological responses to assess their effects on fitness raises further unanswered questions. Linking physiological responses and fitness is likely impossible without establishing response phenotypes, as selection acts on the phenotype (Falconer & Mackay, 1996). Physiological responses are often labile (e.g. White, Schimpf & Cassey, 2012). Therefore, a single measurement may not be representative of an individual's phenotype. Consequently, the lack of within‐individual response repeatability estimates among included studies is a considerable void in our understanding. Not only is it unclear how repeatable thermal responses are (and so how their phenotypes are expressed), but the relationship between thermal response repeatability and that of inferred physiological processes is also unknown. The frequent lability of physiological responses, and the likelihood they involve non‐monotonic physiological fluctuations over time, suggests that specialist approaches to phenotype characterisation such as reaction norms (Hau & Goymann, 2015) and profile repeatability (Reed, Harris & Romero, 2019) may be required to address this knowledge shortfall. Equally, heritability estimates (derived in part from repeatability) could usefully shed light on capacity for intergenerational adaptation. Estimates of adaptation could be obtained through a thermal response function (if the function is shown to be more than a simple byproduct of other processes), or by using the thermal response as a proxy for the inferred physiological process, if the two are sufficiently phenotypically equivalent.

The general paucity of research conducted on wild individuals likely reflects both difficulties inherent in inducing and measuring (non‐surface temperature) physiological responses in natural environments (to validate surface temperature measures), and the agricultural focus of many included studies. However, at least some bias towards controlled environments may relate to technical challenges connected with thermal imaging in natural environments. The aforementioned benefits of thermal imaging (see Section I) are not always entirely straightforward to exploit in such situations. For example, imaging outdoors exposes measurements to several sources of additional thermal noise, including solar radiation, environmental temperature, wind, and humidity fluctuations. Measurement error from the latter three can be largely eliminated by placing an object of known temperature and similar emissivity to the target surface (e.g. a temperature probe) within the field of view, against which the whole image can be calibrated (a simplified version of methods presented by Simpson *et al*., 2008). But the energy input from solar radiation can be more difficult to address, especially when attempting to estimate metabolic heat flux (McCafferty, 2007; Tattersall, 2016). Options such as shielding subjects from direct sunlight, or assessing irradiation levels close to the site of surface temperature measurements (to include in analyses) can only be implemented in situations where subjects visit a known location (e.g. nests or artificial feeding sites). Direct sunlight can also be avoided or minimised more opportunistically by imaging under natural shade (e.g. for forest‐dwelling species), overcast skies, or at night, although such restrictions are undoubtedly limiting. To this end, new approaches facilitating subject irradiation estimation are required to broaden the applicability of thermal imaging under direct sunlight.

Despite these challenges, it is striking that the literature does support some generalised responses in some physiological contexts, as summarised in Fig. 5. Experiments to date indicate that there are minimal exceptions to the expectation that body surface temperatures should rise with environmental temperatures (Fig. 5A). Equally, decreases in metabolism appear to result consistently in decreases in body temperature (Fig. 5B). But perhaps the strongest conclusion from this review is that acute stress results in a highly generalised rapid short‐term decrease in body surface temperature (Fig. 5C). Whereas, it appears likely that body surface temperature responses during immune activation (Fig. 5D) may only be generalisable within discrete ranges of environmental conditions. Further work that is better designed to address the weaknesses in the literature identified above will help confirm and refine these findings.

Assuming knowledge gaps can be filled and technical challenges overcome, inferring physiology in the wild using thermal imaging will present a host of valuable eco‐evolutionary research opportunities surpassing those available with invasive or integrating techniques. As well as helping link physiological phenotypes to fitness, and so potentially allowing population dynamics to be predicted from individual physiological responses, practically unlimited potential for repeated measures is ideal for longitudinal research. Specifically, long‐term monitoring could follow the ontogeny and senescence of coping ability across life stages to establish comprehensive lifelong physiological phenotypes integrating related plasticity (Clutton‐Brock & Sheldon, 2010). Also, the ability to assess changes in physiological responses to repeated challenges over varying timescales is especially well suited to validation of conceptual/mathematical representations of resilience, including the reactive scope model (Wright *et al*., 2023). Such validation would be invaluable for comparative studies establishing when responses transition from beneficial and adaptive to pathological. In addition, effects of thermoregulatory, metabolic, stress and immune challenges on other labile aspects of performance (e.g. cognition) could be usefully investigated over the course of both natural and anthropogenic environmental change.

## V. CONCLUSIONS

(1) Consensus profiling suggests unambiguous thermoregulatory, metabolic and acute stress (up to 3 min from stressor onset) body surface temperature responses are likely to be broadly generalisable across settings and populations in wild birds and mammals.
(2) Chronic stress effects on body surface temperatures have not been studied enough to draw conclusions about their generalisability.
(3) Body surface temperature dynamics during immune activation could also be generalisable, but probably only within discrete ranges of environmental conditions (e.g. below *versus* within *versus* above the subject's thermoneutral zone).
(4) Mechanistic understanding of the processes linking thermoregulatory, metabolic, stress and immune physiology with body surface temperatures remains insufficient. Therefore, those wishing to develop methods for inferring physiology from body surface temperatures in natural environments should prioritise combinations of detailed laboratory validations and confirmatory field studies.
(5) The development of such methods would also benefit from greater rigour than is evident in the currently available literature, in terms of routinely validating physiological challenges, avoiding use of stress‐inducing methods, analysing life‐history stage and sex differences, investigating effects of both challenge increase and decrease, and assessing responses across all possible thermoregulatory states.

## Supporting information


**Appendix S1.** Literature search term specification.
**Fig. S1**. Flow diagram of the systematic review process.
**Table S1.** Numbers of included studies, categorised by functional group (A, thermoregulation; B, metabolism; C, stress; D, immune response), whether physiological challenges were validated or not, and outcome.
**Table S2.** Numbers of included studies, categorised by functional group (A, thermoregulation; B, metabolism; C, stress; D, immune response), the potential for methods to introduce a confounding thermal stress response (high, low or unclear, see Table 2), and outcome.
**Table S3.** Numbers of included studies, categorised by environmental conditions relative to subject thermoneutral zone (TNZ) (below, within or above, where TNZ data were available), and outcome.

**Database S1.** Excel file containing separate tabs for each stage of the systematic review process: the initial search, selection for inclusion, and data extraction for the four physiological functional groups.
