# Supplementary material for: Body surface temperatures as biomarkers of physiological environmental adaptation in wild birds and mammals
Source: Biol Rev Camb Philos Soc. 2025 Oct 2;101(1):336–63. doi: 10.1111/brv.70085 (PMC12783446; doi:10.1111/brv.70085)
Supplement: Supplementary file 1 — Appendix S1. Literature search term specification. Fig. S1. Flow diagram of the systematic review process. Table S1. Numbers of included studies, categorised by functional group (A, thermoregulation; B, metabolism; C, stress; D, immune response), whether physiological challenges were validated or not, and outcome. Table S2. Numbers of included studies, categorised by functional group (A, thermoregulation; B, metabolism; C, stress; D, immune response), the potential for methods to introduce a confounding thermal stress response (high, low or unclear, see Table 2), and outcome. Table S3. Numbers of included studies, categorised by environmental conditions relative to subject thermoneutral zone (TNZ) (below, within or above, where TNZ data were available), and outcome. [file BRV-101-336-s001.docx]

**Appendix S1. Literature search term specification**

Using the *Web of Science* Core Collection, we performed an initial search using the following terms:

TS=(mammal* OR bird*) AND TS=(physiolog* OR thermoregulat* OR metabol* OR stress* OR immun*) AND (TS=(surface* OR peripheral* OR skin*) AND TS=(temperature*))

Within the terms, * represents a truncation wildcard symbolising any group of characters, including no character. We then used litsearchr v1.0 (Grames *et al*., 2019) to extend the initial search algorithm with synonymous search terms to limit bias against unfamiliar studies. Suggested additional search terms (above a cutoff of the lowest of 5 ﻿keyword co‐occurrence network nodes) were manually edited into one of four concept groups: (1) *reject* – not judged specifically relevant to research question; (2) *population* – endotherm species or groups; (3) *physiology* – any reference to physiological processes, including references to internal temperature; and (4) *outcome* – any combined reference to temperature and body surface, plus non-linked references to body surface integuments (e.g. skin, hair, fur, etc.). These concept groups were then used to generate the final search term from which our database was generated:

TS=(aves OR avian OR bat OR bird* OR broiler* OR chick* OR duck* OR endotherm OR hens OR "laying hen*" OR mammal* OR mice OR mouse OR passerine OR penguin* OR poultry OR primate* OR rabbit* OR rat OR rodent* OR seabird* OR seal OR bovine OR "ground squirrel*" OR ovine OR "small mammal*" OR squirrel* OR "terrestrial mammal*") AND TS=("binding protein*" OR "body condition" OR "body mass" OR "body size" OR "body temperature*" OR "circadian clock*" OR "circadian rhythm*" OR "circadian system*" OR circulation OR "cold stress" OR "core body temperature*" OR "core temperature*" OR corticosterone OR "energetic cost*" OR energetics OR "energy budget" OR "energy expenditure" OR "evaporative cooling" OR "evaporative water loss" OR feeding OR fever OR glucocorticoid* OR "heart rate" OR "heat dissipation" OR "heat exchange" OR "heat loss" OR "heat production" OR "heat shock" OR "heat stress" OR "heat transfer" OR heat-stress OR heterothermy OR hibernation OR homeostasis OR hormon* OR hyperthermia OR hypothermia OR hypoxia OR "immune response" OR inflammation OR insulation OR "locomotor activity" OR "metabolic rate*" OR metabolism OR mitochondria OR muscle* OR "oxygen consumption" OR pain OR pathogenesis OR "peripheral clock*" OR "peripheral oscillator*" OR "physiological respons*" OR physiology OR "rectal temperature*" OR respiration OR "resting metabolic rate*" OR scn OR shivering OR "skeletal muscle*" OR sleep OR stress OR "suprachiasmatic nucleus" OR "temperature regulation" OR "thermal conductance" OR "thermal stress" OR thermogenesis OR thermoregulation OR "thermoregulatory respons*" OR torpor OR vasoconstriction OR "activity pattern*" OR adipose OR adrenal OR autonomic OR "basal metabolic" OR breathing OR breeding OR "brown adipose" OR cardiovascular OR "central nervous system" OR "circadian oscillator*" OR cytokine OR endocrine OR endothermic OR "energy balance" OR "energy intake" OR "energy saving" OR "environmental stress") AND (TS=(fur OR hair OR plumage OR skin OR surface OR dermis OR epidermal OR feather OR "peripheral tissue") AND TS=(temperature*))

**Fig. S1.** Flow diagram of the systematic review process.

| **(A) Thermoregulation** |  |  |  |
| --- | --- | --- | --- |
| *Predominant relationship* | **Positive** | **Negative** | **No relationship** |
| Validated | 68(29) | 3(2) | 1(1) |
| Unvalidated | 177(37) | 0(0) | 9(4) |
| *After 15 min (environmental temp. decrease)* | **Increase** | **Decrease** | **No effect** |
| Validated | 3(2) | 12(4) | 1(1) |
| Unvalidated | 0(0) | 12(9) | 1(1) |
| *After 15 min (environmental temp. increase)* | **Above** | **Below** | **No difference** |
| Validated | 19(11) | 2(1) | 0(0) |
| Unvalidated | 14(8) | 0(0) | 0(0) |
| **(B) Metabolism** |  |  |  |
| *Predominant relationship* | **Positive** | **Negative** | **No relationship** |
| Validated | 8(5) | 0(0) | 1(1) |
| Unvalidated | 9(9) | 0(0) | 7(3) |
| *After 15 min (metabolism decrease)* | **Increase** | **Decrease** | **No effect** |
| Validated | 0(0) | 5(2) | 0(0) |
| Unvalidated | 0(0) | 2(2) | 1(1) |
| **(C) Stress** |  |  |  |
| *Predominant relationship* | **Positive** | **Negative** | **No relationship** |
| Validated | 7(4) | 20(7) | 2(1) |
| Unvalidated | 20(9) | 30(19) | 15(12) |
| *Immediate response (stress increase)* | **Increase** | **Decrease** | **No effect** |
| Validated | 0(0) | 11(4) | 0(0) |
| Unvalidated | 3(2) | 16(9) | 6(4) |
| *3 min (stress increase)* | **Increase** | **Decrease** | **No effect** |
| Validated | 0(0) | 8(3) | 0(0) |
| Unvalidated | 6(3) | 9(5) | 4(3) |
| *15 min (stress increase)* | **Increase** | **Decrease** | **No effect** |
| Validated | 3(2) | 4(2) | 1(1) |
| Unvalidated | 5(3) | 3(1) | 4(2) |
| *After 15 min (stress increase)* | **Increase** | **Decrease** | **No effect** |
| Validated | 5(3) | 3(3) | 4(3) |
| Unvalidated | 4(3) | 3(1) | 9(4) |
| **(D) Immune response** |  |  |  |
| *Predominant relationship* | **Positive** | **Negative** | **No relationship** |
| Validated | 8(6) | 18(11) | 5(5) |
| Unvalidated | 10(5) | 2(2) | 8(5) |
| *After 15 min (immune activity increase)* | **Increase** | **Decrease** | **No effect** |
| Validated | 5(5) | 1(1) | 4(3) |
| Unvalidated | 4(2) | 1(1) | 3(2) |

**Table S1.** Numbers of included studies, with the number of papers from which these studies were split in parentheses (see Section II.3), categorised by functional group (A, thermoregulation; B, metabolism; C, stress; D, immune response), whether physiological challenges were validated or not, and outcome. Positive, negative and no relationship outcomes refer to the predominant relationship reported between comparator and body surface temperature across all studies including observational and experimental approaches. Increase, decrease and no effect outcomes are from experimental within-individual comparisons and refer to body surface temperature difference from baseline condition.

| **(A) Thermoregulation** |  |  |  |  |
| --- | --- | --- | --- | --- |
| *Predominant relationship* | **Positive** | **Negative** | **No relationship** | |
| High | 86(26) | 3(2) | 1(1) | |
| Low | 108(18) | 0(0) | 6(2) | |
| Unclear | 51(22) | 0(0) | 3(2) | |
| *After 15 min (environmental temp. decrease)* | **Increase** | **Decrease** | **No effect** | |
| High | 3(2) | 14(6) | 0(0) | |
| Low | 0(0) | 5(2) | 1(1) | |
| Unclear | 0(0) | 5(5) | 1(1) | |
| *After 15 min (environmental temp. increase)* | **Increase** | **Decrease** | **No effect** | |
| High | 17(9) | 2(1) | 0(0) | |
| Low | 6(3) | 0(0) | 0(0) | |
| Unclear | 10(7) | 0(0) | 0(0) | |
| **(B) Metabolism** |  |  |  | |
| *Predominant relationship* | **Positive** | **Negative** | **No relationship** | |
| High | 4(4) | 0(0) | 0(0) | |
| Low | 8(7) | 0(0) | 8(4) | |
| Unclear | 5(2) | 0(0) | 0(0) | |
| *After 15 min (metabolism decrease)* | **Increase** | **Decrease** | **No effect** | |
| High | 0(0) | 2(2) | 0(0) | |
| Low | 0(0) | 1(1) | 1(1) | |
| Unclear | 0(0) | 4(1) | 0(0) | |
| **(C) Stress** |  |  |  | |
| *Predominant relationship* | **Positive** | **Negative** | **No relationship** | |
| High | 12(5) | 11(6) | 9(7) | |
| Low | 13(7) | 35(17) | 6(5) | |
| Unclear | 2(1) | 4(3) | 2(1) | |
| *Immediate response (stress increase)* | **Increase** | **Decrease** | **No effect** | |
| High | 0(0) | 9(5) | 1(1) | |
| Low | 3(2) | 17(7) | 3(2) | |
| Unclear | 0(0) | 1(1) | 2(1) | |
| *3 min (stress increase)* | **Increase** | **Decrease** | **No effect** | |
| High | 0(0) | 5(4) | 2(1) | |
| Low | 6(3) | 12(4) | 2(2) | |
| Unclear | 0(0) | 0(0) | 0(0) | |
| *15 min (stress increase)* | **Increase** | **Decrease** | **No effect** | |
| High | 2(1) | 0(0) | 0(0) | |
| Low | 6(4) | 7(3) | 5(3) | |
| Unclear | 0(0) | 0(0) | 0(0) | |
| *After 15 min (stress increase)* | **Increase** | **Decrease** | **No effect** | |
| High | 3(2) | 0(0) | 6(3) | |
| Low | 6(4) | 6(4) | 7(4) | |
| Unclear | 0(0) | 0(0) | 0(0) | |
| **(D) Immune response** |  |  |  | |
| *Predominant relationship* | **Positive** | **Negative** | **No relationship** | |
| High | 1(1) | 15(9) | 4(4) | |
| Low | 16(9) | 1(1) | 5(3) | |
| Unclear | 1(1) | 4(3) | 4(3) | |
| *After 15 min (immune activity increase)* | **Increase** | **Decrease** | **No effect** | |
| High | 1(1) | 0(0) | 3(2) | |
| Low | 7(5) | 1(1) | 3(2) | |
| Unclear | 1(1) | 1(1) | 1(1) | |

**Table S2.** Numbers of included studies, with the number of papers from which these studies were split in parentheses (see Section II.3), categorised by functional group (A, thermoregulation; B, metabolism; C, stress; D, immune response), the potential for methods used to induce a confounding thermal stress response (high, low or unclear, see Table 2), and outcome. Positive, negative and no relationship outcomes refer to the predominant relationship reported between comparator and body surface temperature across all studies including observational and experimental approaches. Increase, decrease and no effect outcomes are from experimental within-individual comparisons and refer to body surface temperature difference from baseline condition.

| **Immune response** |  |  |  |
| --- | --- | --- | --- |
| *Predominant relationship* | **Positive** | **Negative** | **No relationship** |
| Below | 5(3) | 0(0) | 3(2) |
| Within | 1(1) | 2(2) | 2(2) |
| Above | 2(2) | 1(1) | 1(1) |
| *After 15 min (immune activity increase)* | **Increase** | **Decrease** | **No effect** |
| Below | 4(2) | 0(0) | 1(1) |
| Within | 1(1) | 1(1) | 1(1) |
| Above | 2(2) | 0(0) | 2(1) |

**Table S3.** Numbers of included immune studies, with the number of papers from which these studies were split in parentheses (see Section II.3), categorised by environmental conditions relative to subject thermoneutral zone (TNZ) (below, within or above, where TNZ data were available), and outcome. Positive, negative and no relationship outcomes refer to the predominant relationship reported between comparator and body surface temperature across all studies including observational and experimental approaches. Increase, decrease and no effect outcomes are from experimental within-individual comparisons and refer to body surface temperature difference from baseline condition.
